# Sclerostin expression in trabecular bone is downregulated by osteoclasts

**DOI:** 10.1038/s41598-020-70817-1

**Published:** 2020-08-13

**Authors:** Masanori Koide, Teruhito Yamashita, Kohei Murakami, Shunsuke Uehara, Keigo Nakamura, Midori Nakamura, Mai Matsushita, Toshiaki Ara, Hisataka Yasuda, Josef M. Penninger, Naoyuki Takahashi, Nobuyuki Udagawa, Yasuhiro Kobayashi

**Affiliations:** 1 Division of Hard Tissue Research, Institute for Oral Science, Matsumoto Dental University, 1780 Gobara, Hiro-oka, Shiojiri, Nagano 399-0781 Japan; 2 Department of Biochemistry, Matsumoto Dental University, 1780 Gobara, Hiro-oka, Shiojiri, Nagano 399-0781 Japan; 3 Department of Pharmacology, Matsumoto Dental University, 1780 Gobara, Hiro-oka, Shiojiri, Nagano 399-0781 Japan; 4 Nagahama Institute for Biochemical Science, Oriental Yeast Co., Ltd., 50 Kano-cho, Nagahama, Shiga 526-0804 Japan; 5 Institute of Molecular Biotechnology of the Austrian Academy of Science (IMBA), Vienna Biocentre, Vienna, Austria; 6 Department of Medical Genetics, Life Science Institute, University of British Columbia, Vancouver, Canada

**Keywords:** Immunochemistry, Biomarkers, Drug development, Experimental models of disease, Genetics research, Calcium and phosphate metabolic disorders, Biomarkers, Biochemistry, Cell biology

## Abstract

Bone tissues have trabecular bone with a high bone turnover and cortical bone with a low turnover. The mechanisms by which the turnover rate of these bone tissues is determined remain unclear. Osteocytes secrete sclerostin, a Wnt/β-catenin signaling antagonist, and inhibit bone formation. We found that sclerostin expression in cortical bone is more marked than in trabecular bone in *Sost* reporter mice. Leukemia inhibitory factor (LIF) secreted from osteoclasts reportedly suppressed sclerostin expression and promoted bone formation. Here, we report that osteoclasts downregulate sclerostin expression in trabecular bone and promote bone turnover. Treatment of C57BL/6 mice with an anti-RANKL antibody eliminated the number of osteoclasts and LIF-positive cells in trabecular bone. The number of sclerostin-positive cells was increased in trabecular bone, while the number of β-catenin-positive cells and bone formation were decreased in trabecular bone. Besides, *Tnfsf11* heterozygous (*Rankl*^+/−^) mice exhibited a decreased number of LIF-positive cells and increased number of sclerostin-positive cells in trabecular bone. *Rankl*^+/−^ mice exhibited a decreased number of β-catenin-positive cells and reduced bone formation in trabecular bone. Furthermore, in cultured osteoclasts, RANKL stimulation increased *Lif* mRNA expression, suggesting that RANKL signal increased LIF expression. In conclusion, osteoclasts downregulate sclerostin expression and promote trabecular bone turnover.

## Introduction

Osteoclastic bone resorption followed by osteoblastic bone formation repeats under physiological conditions and maintains both blood calcium levels and bone homeostasis^1,2^. Receptor activator of NF-κB ligand (RANKL) and osteoprotegerin (OPG), a decoy receptor of RANKL, regulate osteoclast differentiation. Both of these are produced by osteoblast-lineage cells such as osteoblasts and osteocytes^3,4^. RANKL binds to its receptor RANK and induces osteoclast development. On the other hand, OPG inhibits the RANKL-RANK interactions and then suppresses osteoclast differentiation. *Tnfrsf11b*-deficient (*Opg*^–/–^) mice exhibited severe osteopenia with enhanced bone resorption and formation^5,6^. The administration of bisphosphonates or an anti-RANKL neutralizing antibody, antiresorptive agents, to *Opg*^–/–^ mice suppressed bone resorption, and subsequently suppressed the enhanced bone formation^7,8^. Clinical studies reported that denosumab, an anti-RANKL antibody, suppressed bone turnover in postmenopausal women^9,10^. Thus, bone formation is coupled with bone resorption during bone remodeling.

Sclerostin (encoded by the *Sost* gene), a protein secreted primarily by osteocytes, is an antagonist of Wnt/β-catenin signaling that inhibits bone accrual^11,12^. Loss of function mutation of the *Sost* gene caused sclerosteosis with abnormally increased bone mass^13^. The mutation caused hypertrophy of cranial and cortical bone, and syndactyly^14^. *Sost*-knock-out (*Sost*^–/–^) mice demonstrated thickening of cranial bone and cortical bone due to increased bone formation^15^. The treatment with an anti-sclerostin neutralizing antibody has been reported to increase bone mass with increased bone formation^16,17^. These findings indicate that the reduction of sclerostin expression increases bone formation.

Sclerostin expression is reportedly inhibited by prostaglandin E_2_ (PGE_2_), parathyroid hormone (PTH), and IL-6 family members such as cardiotrophin-1 (CT-1), oncostatin M (OSM), and leukemia inhibitory factor (LIF)^8,18–21^. Administration of PTH, CT-1, OSM, and LIF accelerated osteogenesis in vitro and in vivo^18,20–23^. The role of PTH in bone acquisition is thought to be partly mediated by the suppression of sclerostin expression^24,25^. Furthermore, the glycoprotein 130 (gp130) signal by CT-1, OSM, and LIF suppressed sclerostin expression in UMR106 cells^20^. We demonstrated that osteoclast-secreted LIF suppressed sclerostin expression and accelerated bone formation in vitro and in vivo^8^. Accordingly, sclerostin expression in osteocytes is controlled by several factors to promote bone turnover.

Bone tissues are composed of mesh-like trabecular bone and a hard outer layer of cortical bone. The trabecular bone was remodeled at a high turnover rate that was likely affected by alterations in the Wnt/β-catenin signaling pathway^26^. However, it is unclear how bone turnover is controlled. Sclerostin expression in cortical bone is reportedly more marked than that in trabecular bone^27^. We reported that *Opg*^−/−^ mice with enhanced bone resorption had reduced sclerostin expression in osteocytes^8^. In contrast, the administration of an antiresorptive agent to the mice increased sclerostin expression and reduced bone formation. Furthermore, osteoclast-secreted LIF suppressed sclerostin expression^8^. Moreover, osteoclasts were frequently observed in trabecular bone compared to in cortical bone. Therefore, we hypothesized that osteoclasts downregulate sclerostin expression in trabecular bone and thereby accelerate bone formation.

Here, we found that the treatment of C57BL/6 mice with an anti-RANKL antibody upregulated sclerostin expression in trabecular bone and reduced the number of osteoclasts. Moreover, treatment with the anti-RANKL antibody reduced the number of β-catenin-positive cells and bone formation in trabecular bone. Furthermore, *Tnfsf11* heterozygous (*Rankl*^+/−^) mice, in which bone resorption is suppressed, exhibited increased numbers of sclerostin-positive cells in trabecular bone. *Rankl*^+*/−*^ mice also had fewer β-catenin-positive cells and suppressed bone formation in trabecular bone. Thus, the suppression of sclerostin expression by osteoclasts plays a critical role in bone formation in trabecular bone.

## Results

### Localization of sclerostin-positive osteocytes and osteoclasts in bone tissues

**Figure 1:**
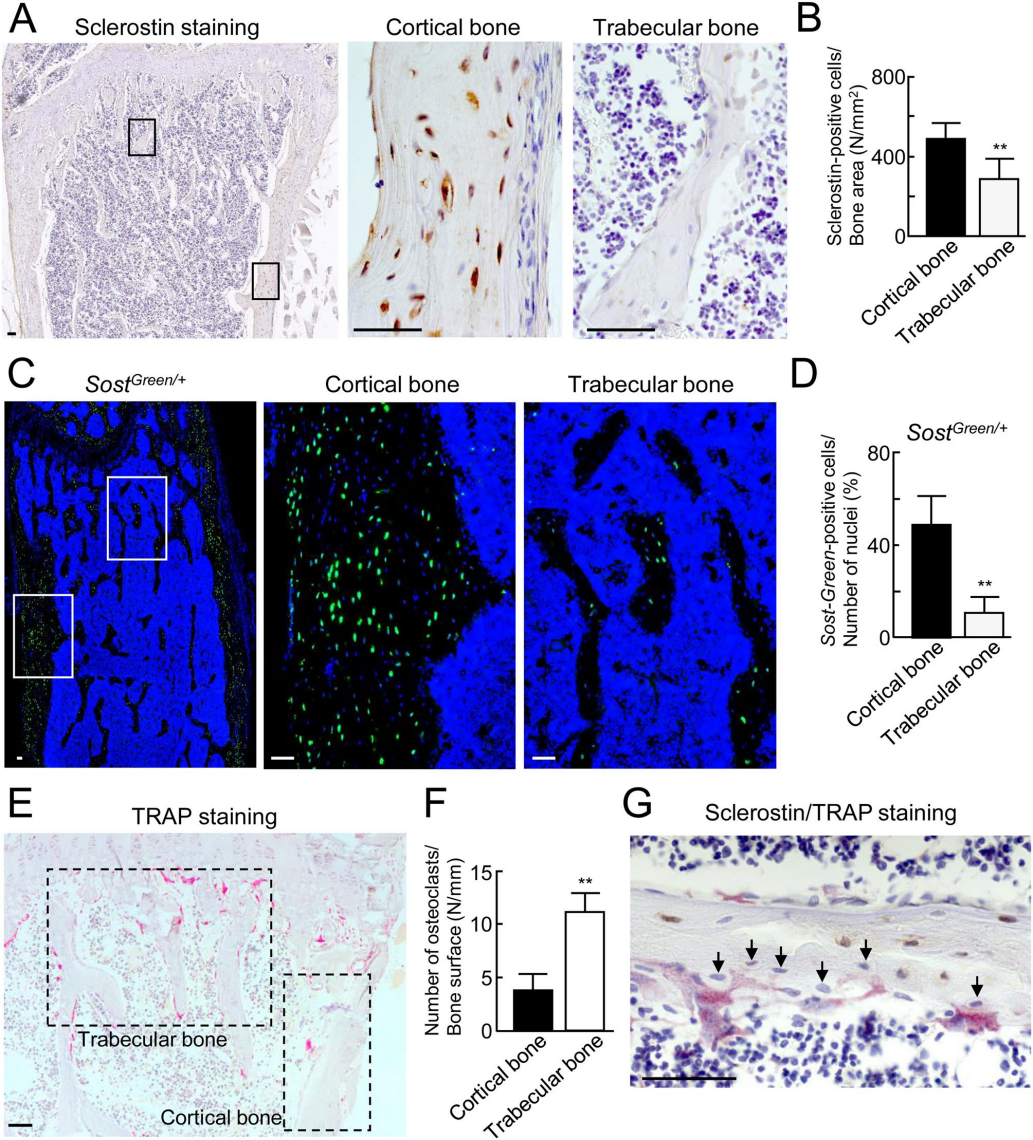
Localization of sclerostin-positive osteocytes and osteoclasts in trabecular bone. (**A**) Immunohistochemical analysis of sclerostin in the femora from C57BL/6 male mice at 12 weeks of age (left panel). The boxed areas in the left panel show the high-power field of cortical and trabecular areas. The center panel shows the cortical areas. The right panel shows the trabecular areas. Sclerostin-positive signals (brown). (**B**) Sclerostin-positive cells/bone area (N/mm^2^). (*n* = 5). (**C**) Histochemical analysis of *Sost-Green* in the femora from 12-week-old *Sost*^*Green/*+^ knock-in male mice (left panel). The boxed areas in the left panel shows the high-power field of cortical and trabecular areas. *Sost*-positive signals were observed (green). Nuclei were stained with Hoechst 33342 (blue). (**D**) *Sost-Green*-positive cells/number of nuclei (%). (*n* = 5). (**E**) Histochemical analysis of TRAP in the tibiae of C57BL/6 male mice at 12 weeks of age. TRAP-positive cells (red). The ROI are indicated by the areas surrounded by the broken lines. (**F**) Number of osteoclasts/bone surface (N/mm) (*n* = 6). (**G**) Double staining of sclerostin and TRAP in the trabecular area of tibiae from C57BL/6 male mice at 12 weeks of age. Black arrows marked sclerostin-negative osteocytes. **p < 0.01. Scale bar, 50 μm.

We compared the localization of sclerostin-positive osteocytes in trabecular bone with that in cortical bone in femora of C57BL/6 male mice at 12 weeks of age using immunohistochemical analyses. Sclerostin expression in osteocytes of trabecular bone was lower compared to that in those of cortical bone (Fig. 1A,B). Furthermore, we generated *Sost*^*Green/*+^ mice in which green fluorescent protein was knocked-in to report the expression of the *Sost* gene (Supplementary Fig. S1A,B). Expression of *Sost-Green*-positive osteocytes was hardly detected in bony areas during embryonic development (Supplementary Fig. S2A–C). Expression of *Sost-Green*-positive osteocytes was detected in the cortical bone from 3 days after birth (Supplementary Fig. S2D–G). The number of *Sost-Green*-positive osteocytes was significantly increased by four-fold in cortical bone of *Sost*^*Green/*+^ male mice at 12 weeks of age as compared to trabecular bone (Fig. 1C,D). Sclerostin expression is negatively controlled by several factors such as bone age, mechanical loading, PTH, and IL-6 family cytokines. We previously reported that LIF derived from osteoclasts suppressed sclerostin expression in osteocytes^8^. We compared the localization of osteoclasts in trabecular bone with those in cortical bone. Many TRAP-positive osteoclasts were present on the surface of trabecular bone as compared with those of cortical bone (Fig. 1E,F). In addition to this, double-staining of sclerostin and TRAP revealed that no sclerostin-positive osteocytes were present near TRAP-positive osteoclasts in trabecular bone (Fig. 1G, arrows). These data indicate that osteoclasts may suppress sclerostin expression in trabecular bone.

### Impacts of the elimination of bone resorption on LIF expression in trabecular bone

**Figure 2:**
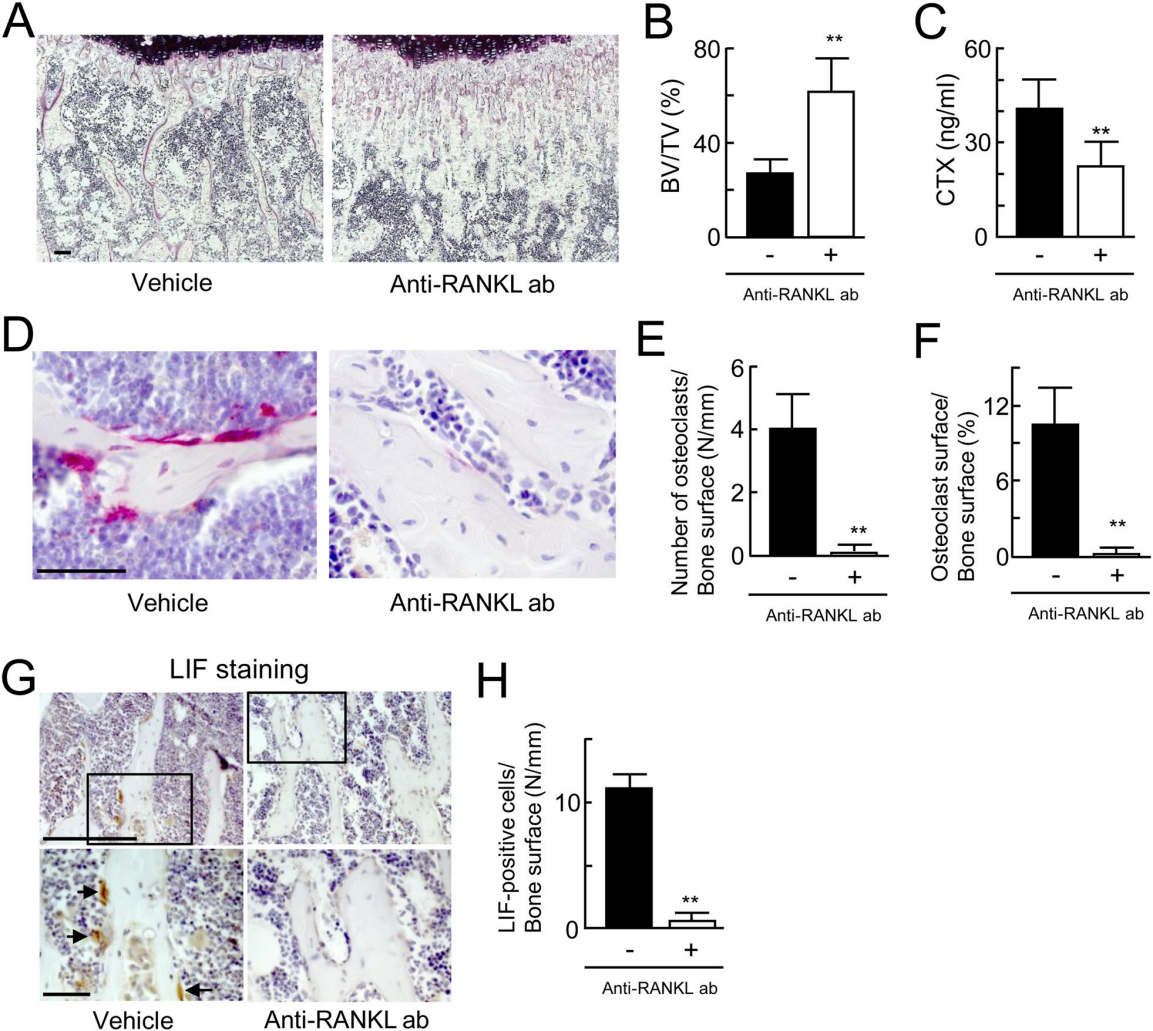
Impacts of the elimination of on bone resorption and LIF expression in C57BL/6 mice. (**A**) Histochemical analysis of the trabecular area of tibiae from C57BL/6 male mice at 12 weeks of age injected with vehicle (left panel) or the anti-RANKL antibody (right panel). (**B**) Trabecular bone volume of the tibiae (*n* = 6). (**C**) Levels of CTX in serum from C57BL/6 mice injected with vehicle or the anti-RANKL antibody (*n* = 6). (**D**) Histochemical studies of TRAP in the trabecular area of the tibiae from C57BL/6 mice injected with vehicle (left panel) or the anti-RANKL antibody (right panel). TRAP (red). (**E**, **F**) Number of osteoclasts/bone surface (N/mm) (*n* = 6). Osteoclast surface/bone surface (%) (*n* = 6). (**G**) Immunohistochemical analysis of LIF in the trabecular area of the femur from C57BL/6 mice injected with vehicle (left panels) or the anti-RANKL antibody (right panels). (**H**) LIF-positive cells/bone surface (N/mm). (*n* = 6). **p < 0.01. Scale bar, 50 μm.

In order to evaluate whether LIF expression is suppressed by the elimination of bone resorption, we examined the LIF expression in trabecular bone of C57BL/6 male mice injected with the anti-RANKL antibody. Treatment of C57BL/6 mice with the anti-RANKL antibody (5 mg/kg) reduced the number of osteoclasts in femurs on day 4^28^. The antibody was injected once s.c. into the mice at 10 weeks of age. Two weeks after administration of the antibody, tibiae and femora were collected and evaluated. Histomorphometric analysis demonstrated that the primary trabecular bone volume was markedly increased in the mice injected with the antibody as compared to vehicle treatment (Fig. 2A,B). The serum CTX levels, a marker of bone resorption, were moderately lower in the mice injected with the antibody (Fig. 2C). The number of TRAP-positive osteoclasts was much lower in the mice injected with the antibody (Fig. 2D,E). The osteoclast surface area was also significantly smaller in the mice injected with the anti-RANKL antibody (Fig. 2F). These data demonstrate that injection of the anti-RANKL antibody markedly suppresses both the differentiation and bone resorption of osteoclasts.

On immunohistochemical staining of LIF in femora of vehicle-injected C57BL/6 mice, LIF-positive cells were observed on trabecular bone (Fig. 2G,H). These large cells, indicated by arrows, are probably osteoclasts. LIF-positive signals were weak in mononuclear cells on bone surfaces including osteoblasts in wild-type C57BL/6 male mice at 12 weeks of age. However, LIF-positive cells were rarely observed in the mice injected with the anti-RANKL antibody. These findings indicate that the anti-RANKL antibody reduced LIF-positive osteoclasts in trabecular bone.

### Impacts of the anti-RANKL antibody on sclerostin expression in trabecular bone

**Figure 3:**
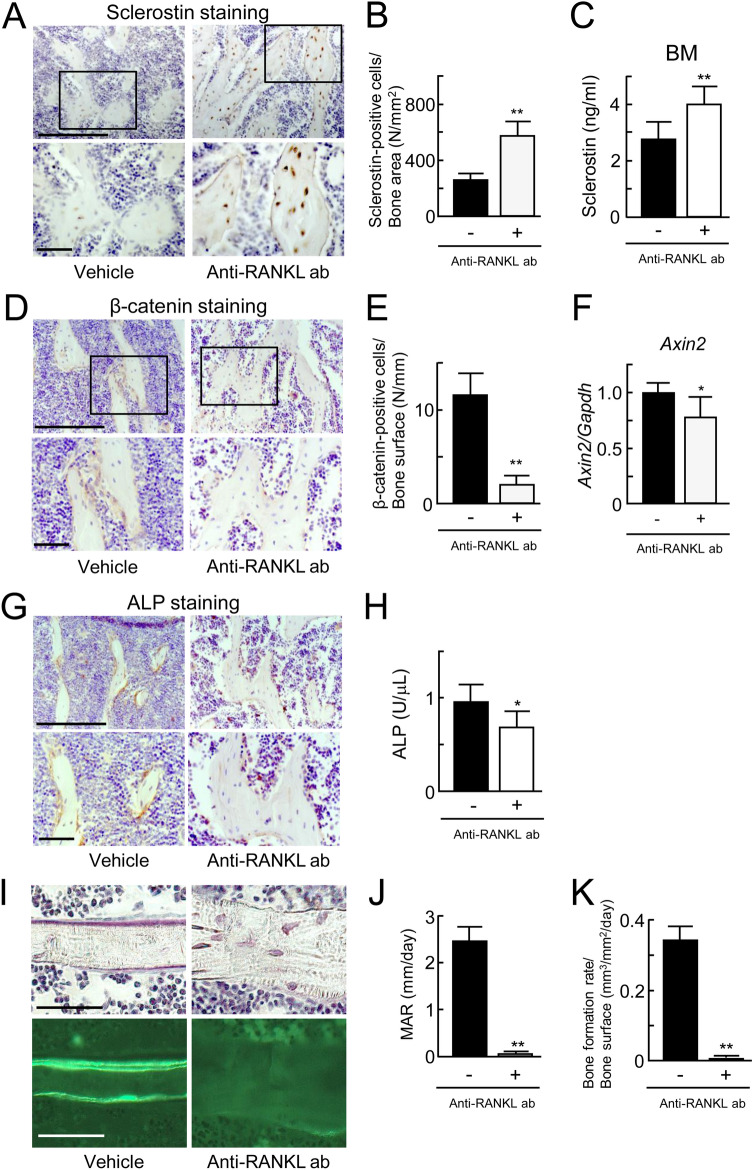
Expression of sclerostin, β-catenin, and ALP in the anti-RANKL antibody-treated C57BL/6 mice. (**A**) Immunohistochemical analysis of sclerostin in the trabecular area of the femur from C57BL/6 mice at 12 weeks of age injected with vehicle (left panels) or the anti-RANKL antibody (right panels). (**B**) Sclerostin-positive cells/bone area (N/mm^2^). (*n* = 6). (**C**) Measurement of sclerostin in bone marrow aspirates from C57BL/6 mice injected with vehicle or the anti-RANKL antibody using ELISA (*n* = 6). (**D**) Immunohistochemical analysis of β-catenin in the trabecular area of the femur from C57BL/6 mice injected with vehicle (left panels) or the anti-RANKL antibody (right panels). (**E**) β-catenin-positive cells/bone surface (N/mm). (*n* = 6). (**F**) Analysis of *Axin2* mRNA expression in tibiae of C57BL/6 mice at 12 weeks of age injected with vehicle or the anti-RANKL antibody using real-time RT-PCR (*n* = 6). (**G**) Immunohistochemical analysis of ALP in the trabecular area of the femur from C57BL/6 mice injected with vehicle (left panel) or the anti-RANKL antibody (right panel). (**H**) ALP activity in serum from C57BL/6 mice injected with vehicle or the anti-RANKL antibody (*n* = 6). (**I**) Histological analysis of Villanueva Goldner staining in the trabecular area of the tibiae from C57BL/6 mice injected with vehicle (top left panel) or the anti-RANKL antibody (top right panel). The lower two panels show double labeling of calcein. (**J**) Bone histomorphometry analysis of mineral apposition rate (MAR) in the tibiae from C57BL/6 mice injected with vehicle (left panel) or the anti-RANKL antibody (right panel). (*n* = 6). (K) Bone histomorphometry analysis of bone formation rate (BFR) in the tibiae from C57BL/6 mice injected with vehicle (left panel) or the anti-RANKL antibody (right panel) (*n* = 6). *p < 0.05, **p < 0.01. Scale bar, 50 μm.

Walker et al.^20^ and we^8^ previously reported that treatment with recombinant LIF restrained sclerostin expression in osteocyte-like cells. Therefore, we investigated whether the reduction of LIF expression by treatment with the anti-RANKL antibody elevates the area of sclerostin expression in trabecular bone using immunohistochemical staining. The number of sclerostin-positive osteocytes was significantly elevated by two-fold in the trabecular bone of the mice injected with the anti-RANKL antibody (Fig. 3A,B). Sclerostin-positive signals in immature osteocytes of primary spongy bone were also increased in the mice injected with the anti-RANKL antibody (Supplementary Fig. S3). We previously reported that the protein level of sclerostin in bone marrow aspirates from C57BL/6 mice was about several tens of times higher than that in serum^8^. Thus, we assessed the amount of sclerostin in bone marrow aspirates from the mice injected with the anti-RANKL antibody or vehicle (Fig. 3C). The amount of sclerostin in bone marrow aspirates was significantly higher in the mice injected with the antibody than in those injected with the vehicle. In contrast to the increased sclerostin-positive osteocytes, the number of β-catenin-positive cells on the surface of trabecular bone was significantly decreased to 20% in the mice injected with the antibody (Fig. 3D,E). Furthermore, *Axin2* expression level was evaluated as a target of Wnt/β-catenin signaling. Treatment with anti-RANKL antibody significantly reduced *Axin2* mRNA expression in the whole tibiae (Fig. 3F). These findings suggest that the anti-RANKL antibody suppressed the Wnt/β-catenin signaling corresponding to the upregulation of sclerostin expression in trabecular bone and bone marrow.

We examined whether the increased sclerostin expression by treatment with the anti-RANKL antibody affects bone formation in trabecular bone (Fig. 3G–K). On immunohistochemical staining for alkaline phosphatase (ALP), ALP-positive signals in the cells of trabecular bone were reduced in the mice injected with the antibody (Fig. 3G). The serum ALP activity was also reduced in the mice injected with the antibody (Fig. 3H). Histomorphometric analysis revealed that both the mineral apposition rate (MAR) and bone formation rate (BFR), indicators of bone-forming activity, in trabecular bone were almost completely abrogated in the mice injected with the antibody (Fig. 3I–K). These data suggest that the anti-RANKL antibody downregulated bone formation activity in trabecular bone corresponding to the increased expression of sclerostin.

### Downregulation of bone resorption and the expression of LIF in *Rankl*^+/−^ mice

To further examine the role of osteoclasts in the downregulation of sclerostin, we focused on *Rankl*^−/−^ mice in which osteoclast formation was completely inhibited^29^. The femur of *Rankl*^−/−^ male mice at 12 weeks of age was filled with primary trabecular bone, and the bone marrow cavity was not observed. The trabecular bone in *Rankl*^−/−^ mice was calcified bone rich in cartilage matrix stained with alcian blue or toluidine blue (Supplementary Fig. S4A,B). Immunohistological analysis of dentin matrix protein-1 (DMP-1) and fibroblast growth factor-23 (FGF-23), important markers of osteocytes, in the femur of *Rankl*^−/−^ mice was also performed. DMP-1- or FGF-23-positive osteocytes in trabecular bone of *Rankl*^−/−^ mice were markedly reduced as compared with wild-type (WT) mice (Supplementary Fig. S4C,D). These data indicate that the features of osteocytes in trabecular bone of *Rankl*^−/−^ mice are markedly different from those of WT mice.

**Figure 4:**
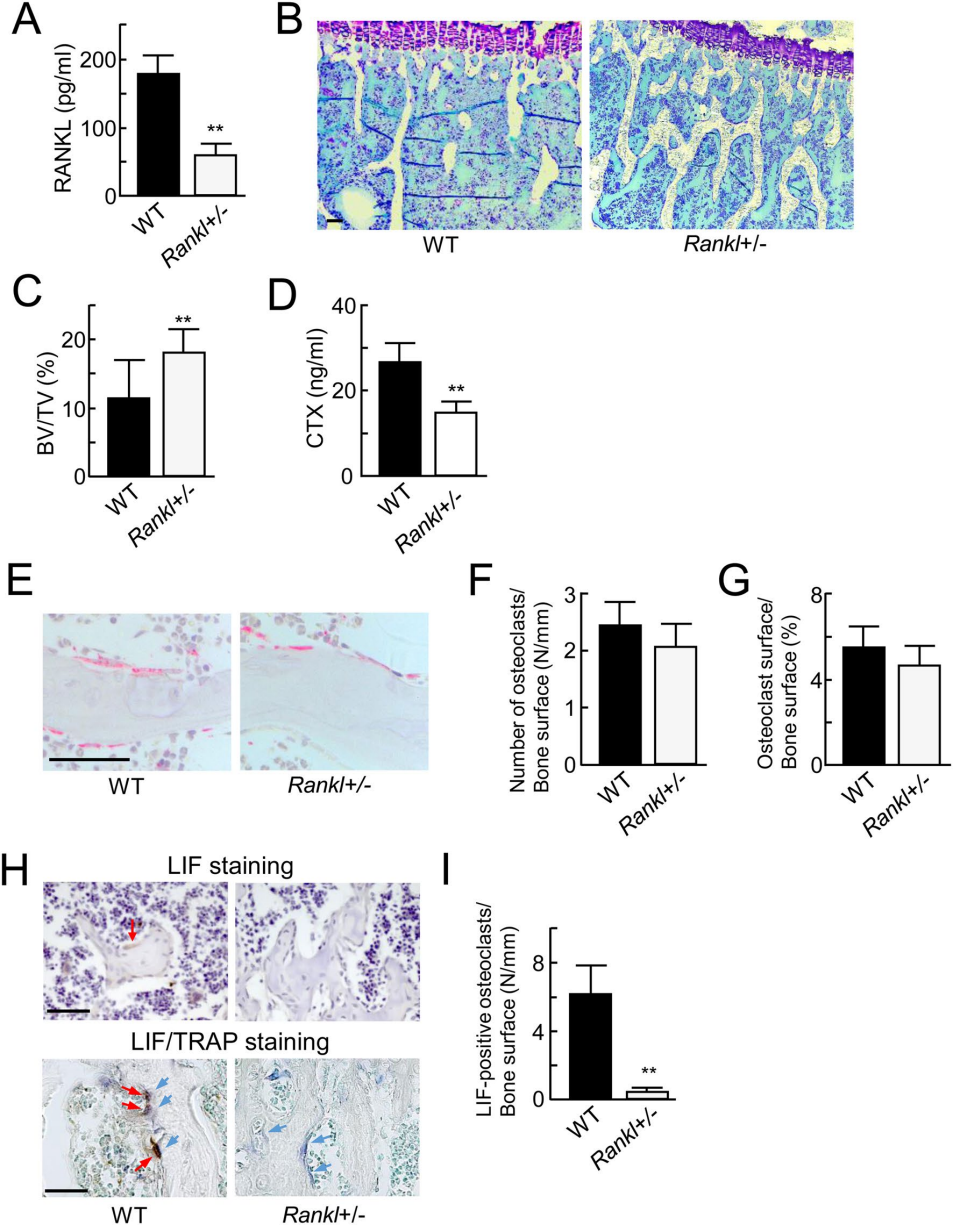
Bone resorption and LIF expression in *Rankl*^+/−^ mice. (**A**) ELISA analysis of RANKL in serum from WT and *Rankl*^+/−^ male mice at 12 weeks of age (*n* = 5). (**B**) Histological analysis of toluidine blue staining in the trabecular area of the tibiae from WT mice (left panel) and *Rankl*^+/−^ mice (right panel). (**C**) Trabecular bone volume of tibiae from WT mice and *Rankl*^+/−^ mice (*n* = 5). (**D**) Levels of CTX in serum from WT and *Rankl*^+/−^ mice (*n* = 5). (**E**) Histochemical analysis of TRAP in the trabecular area of the tibiae from WT mice (left panel) and *Rankl*^+/−^ mice (right panel). TRAP (red). (**F**) Number of osteoclasts/bone surface (N/mm) (*n* = 5). (**G**) Osteoclast surface/bone surface (%) (*n* = 5). (**H**) Immunohistochemical analysis of LIF in the trabecular area of the femur from WT mice (top left panels) and *Rankl*^+/−^ mice (top right panels). The lower two panels show double-staining of LIF and TRAP. LIF-positive cells were marked by red arrows. TRAP-positive cells were marked by blue arrows. (**I**) LIF-positive cells/bone surface (N/mm). (*n* = 5). **p < 0.01. Scale bar, 50 μm.

Therefore, we evaluated bone resorption in 12-week-old *Rankl*^+*/*−^ male mice. RANKL serum levels in *Rankl*^+*/*−^ mice were reduced to 35% as compared with WT mice (Fig. 4A). Histomorphometric analysis demonstrated that the trabecular bone volume in femora was moderately elevated in *Rankl*^+*/*−^ mice as compared to WT mice (Fig. 4B,C), but smaller than that in mice treated with anti-RANKL antibody (see Fig. 2A). The serum levels of CTX were significantly decreased by half in *Rankl*^+/−^ mice (Fig. 4D). By TRAP staining, osteoclasts were observed in trabecular bone. The number of TRAP-positive osteoclasts and osteoclast surface slightly decreased, but not significantly (Fig. 4E–G). The levels of CTX in serum were decreased in *Rankl*^+/−^ mice, suggesting that the bone resorbing activity of osteoclasts was decreased. These data indicated that the trabecular bone volume is increased in *Rankl*^+/−^ mice with the suppression of bone resorption.

On immunohistochemical analysis, LIF-positive signals (indicated by red arrows) were mainly observed in TRAP-positive osteoclasts (indicated by blue arrows) on the trabecular bone surface of WT mice. On the other hand, there were fewer LIF-positive osteoclasts in *Rankl*^+/−^ mice even through the number of osteoclasts remained as well as that in WT mice (Fig. 4F–I). These data suggest that the suppression of bone resorption might reduce LIF expression in osteoclasts of *Rankl*^+/−^ mice.

### Enhancement of sclerostin expression in trabecular bone of *Rankl*^+/−^ mice

**Figure 5:**
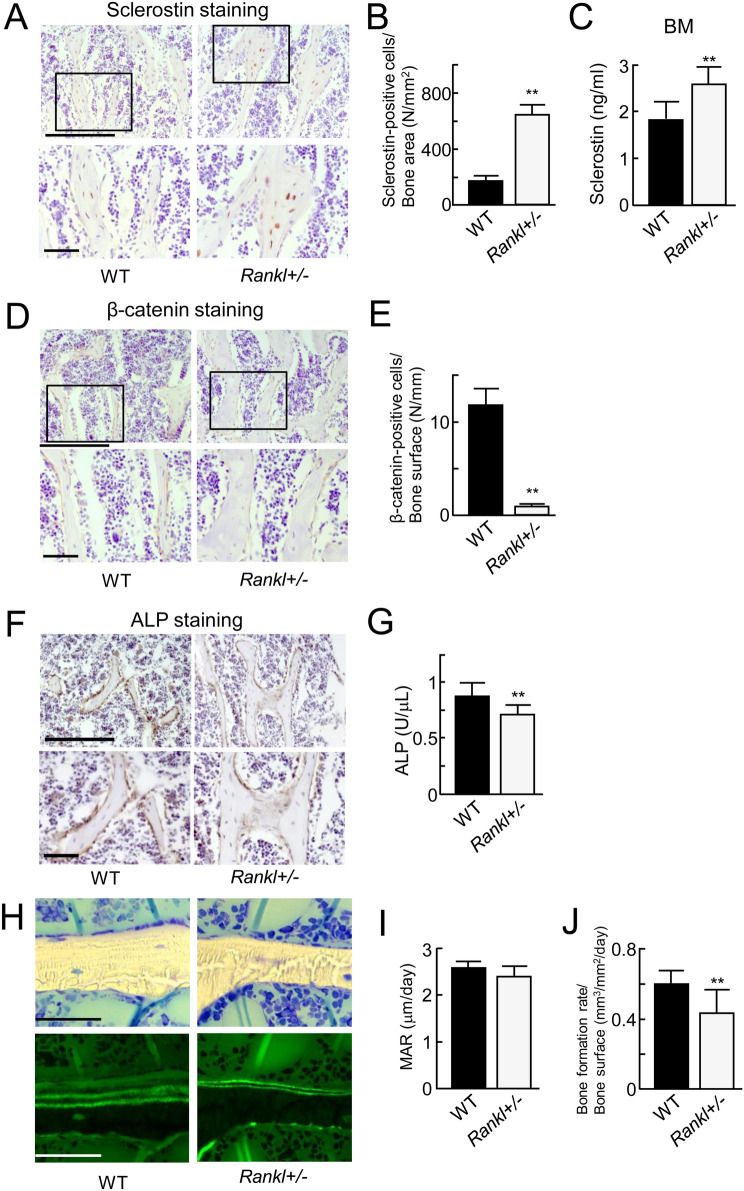
Expression of sclerostin, β-catenin, and ALP in *Rankl*^+/−^ mice. (**A**) Immunohistochemical analysis of sclerostin in the trabecular area of the femur from WT mice at 12 weeks of age (left panels) and *Rankl*^+/−^ mice (right panels). (**B**) Sclerostin-positive cells/bone area (N/mm^2^). (*n* = 5). (**C**) Measurement of sclerostin in bone marrow aspirates from WT mice and *Rankl*^+/−^ mice using ELISA (*n* = 5). (**D**) The expression of β-catenin in the trabecular area of the femur from WT mice (left panels) and *Rankl*^+/−^ mice (right panels). (**E**) β-catenin-positive cells/bone surface (N/mm). (*n* = 5). (**F**) Immunohistochemical analysis of ALP in the trabecular area of the femur from WT mice (left panel) and *Rankl*^+/−^ mice (right panel). (**G**) ALP activity in serum from WT and *Rankl*^+/−^ mice (*n* = 5). (**H**) Histological analysis of toluidine blue staining in the trabecular area of the tibiae from WT mice (top left panel) and *Rankl*^+/−^ mice (top right panel). The lower two panels show double labeling of calcein. (**I**) Bone histomorphometry analysis of mineral apposition rate (MAR) in the tibiae from WT mice (left panel) and *Rankl*^+/−^ mice (right panel). (*n* = 5). (**J**) Bone histomorphometry analysis of bone formation rate (BFR) in the tibiae from WT mice (left panel) and *Rankl*^+/−^ mice (right panel). (*n* = 5). **p < 0.01. Scale bar, 50 μm.

We next investigated sclerostin expression in femora of *Rankl*^+/−^ and WT mice using immunostaining. Elevated sclerostin expression was observed in *Rankl*^+/−^ mice as compared with WT mice. The number of sclerostin-positive osteocytes was significantly increased by three-fold in *Rankl*^+/−^ mice (Fig. 5A,B). Furthermore, ELISA revealed that the amount of sclerostin in bone marrow aspirates moderately increased in *Rankl*^+/−^ mice (Fig. 5C). In contrast to the increased sclerostin-positive osteocytes, the signals of β-catenin-positive cells on trabecular bone were markedly decreased in *Rankl*^+/−^ mice (Fig. 5D,E).

We further explored whether the elevated sclerostin expression in *Rankl*^+/−^ mice affects bone formation in trabecular bone (Fig. 5F–J). On immunohistochemical staining, the ALP-positive signal intensity in trabecular bone was diminished in *Rankl*^+/−^ mice (Fig. 5F). The serum ALP activity was also significantly reduced in *Rankl*^+/−^ mice (Fig. 5G). Histomorphometric analysis demonstrated that the BFR, but not MAR, was significantly decreased in trabecular bone from *Rankl*^+/−^ mice (Fig. 5H–J). These data indicate that sclerostin expression, Wnt/β-catenin signals, and bone formation in trabecular bone of *Rankl*^+/−^ mice exhibit an inverse pattern to those in WT mice.

### Enhancement of LIF expression in macrophages and osteoclasts thorough RANKL signals

**Figure 6:**
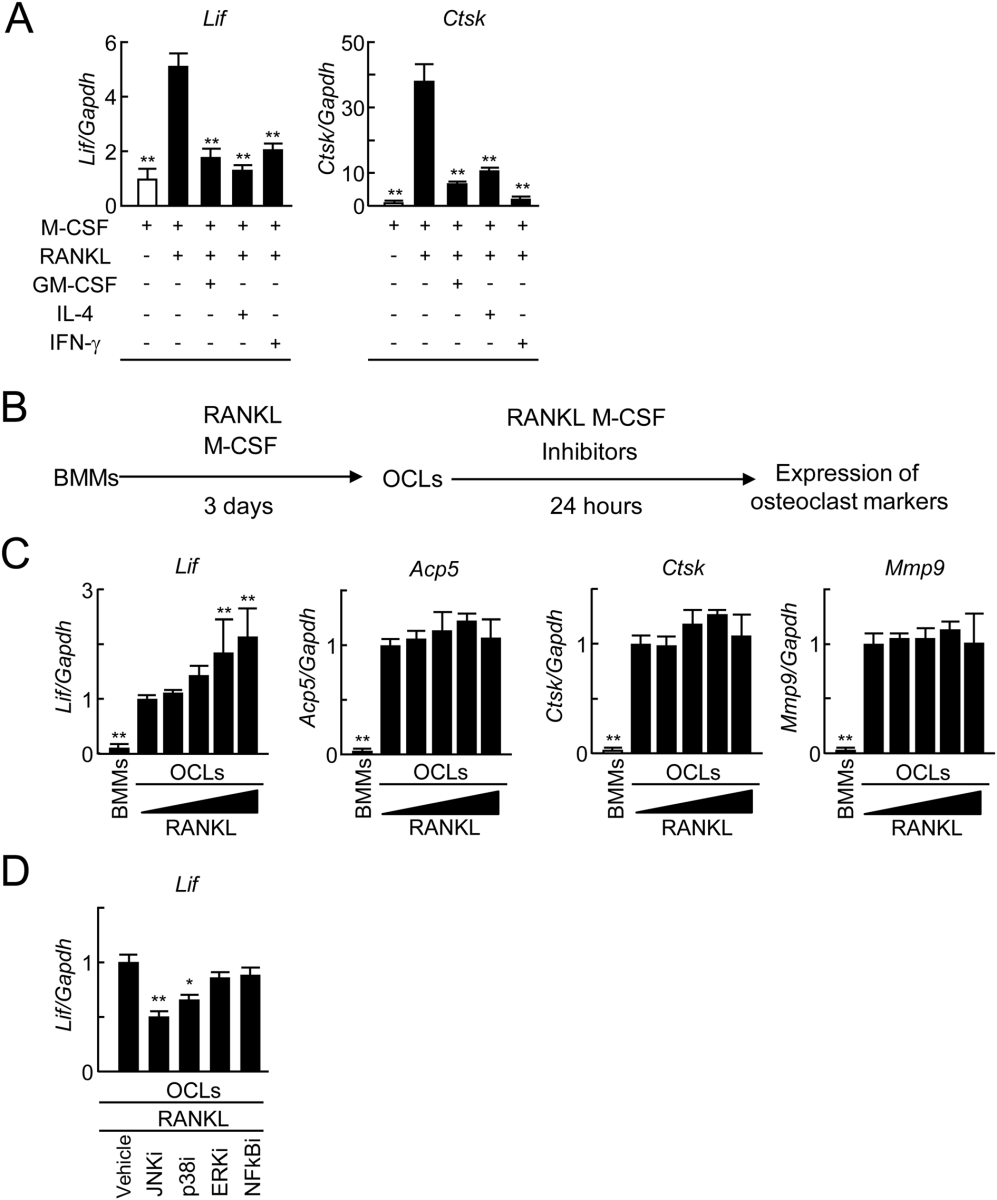
Expression of LIF in RANKL-stimulated BMMs and osteoclasts. (**A**) BMMs were cultured in the presence or absence of GST-RANKL (100 ng/ml), GM-CSF (10 ng/ml), IL-4 (10 ng/ml), or IFN-γ (20 ng/ml) with M-CSF (50 ng/ml) for 24 h. Analysis of the expression of *Lif* and *Ctsk* mRNAs in the cultured BMMs using real-time PCR (*n* = 3). (**B**) BMMs were cultured in the presence or absence of GST-RANKL (100 ng/ml) with M-CSF (50 ng/ml) for 3 days. Osteoclasts (OCLs) appeared on day 3. In addition, the OCLs were further cultured in the presence of varying concentrations of GST-RANKL (0, 1, 5, 25, or 100 ng/ml) with M-CSF (50 ng/ml) for 24 h. For inhibitor experiments, the OCLs were further cultured in the presence or absence of inhibitors of JNK, p38 MAPK, ERK, or NF-κB pathways with GST-RANKL (100 ng/ml) and M-CSF (50 ng/ml) for 24 h. (**C**) Analysis of the expression of *Lif, Acp5, Ctsk,* and *Mmp9* mRNAs in the cultured OCLs in the presence of varying concentrations of GST-RANKL (0, 1, 5, 25, or 100 ng/ml) with M-CSF using real-time PCR (*n* = 4). (**D**) Analysis of the expression of *Lif* mRNA in the cultured OCLs in the presence or absence of inhibitors of JNK (10 μM), p38 MAPK (10 μM), ERK (20 μM), or NF-κB (5 μM) pathways with GST-RANKL (100 ng/ml) and M-CSF using real-time PCR (*n* = 4). *p < 0.05, **p < 0.01.

We have demonstrated that RANKL depletion reduces the number of LIF-positive cells in vivo (Figs. 2H, 4I). Therefore, we examined whether RANKL-induced signals directly regulate the expression of LIF in osteoclast differentiation. Bone marrow macrophages (BMMs) were cultured for 24 h in the presence of M-CSF with or without RANKL. Inhibitors of osteoclast differentiation, granulocyte–macrophage colony-stimulating factor (GM-CSF)^30^, interleukin-4 (IL-4)^31^, and interferon-gamma (IFN-γ)^32^ were added into those cultures. The expression of *Lif* and an osteoclast marker, such as *Ctsk* (encoding cathepsin K) was assessed using real-time PCR. After RANKL stimulation, the expression of *Lif* and *Ctsk* mRNA in BMMs significantly increased within 24 h (Fig. 6A). Treatment with GM-CSF, IL-4 or IFN-γ suppressed RANKL-induced expression of *Lif* and *Ctsk* mRNA in BMMs (Fig. 6A). These results suggested that RANKL signals induced the expression of LIF in BMMs.

Furthermore, we examined whether RANKL-induced signals directly regulate the expression of LIF in osteoclasts. To obtain osteoclasts, BMMs were cultured for 3 days in the presence of M-CSF and RANKL (Fig. 6B). After osteoclasts were formed, varying concentrations of RANKL were added to osteoclast cultures with 50 ng/ml of M-CSF for 24 h. The expression of *Lif* and osteoclast markers, such as *Acp5* (encoding TRAP),* Ctsk,* and *Mmp9* (encoding matrix metalloproteinase 9)*,* was assessed using real-time PCR. RANKL stimulation upregulated *Lif* mRNA expression in a dose-dependent manner (Fig. 6C). On the other hand, the expression of *Acp5*,* Ctsk*, and *Mmp9*, was maintained even in cultures treated with a high concentration of RANKL. These results suggested that RANKL signals increase the expression of LIF in osteoclasts.

Expression of LIF was reportedly induced via the c-Jun N-terminal kinase (JNK) pathway, one of the mitogen-activated protein kinase (MAPK) pathways, by tumor necrosis factor alpha (TNF-α) stimulation in myoblasts^33^. TGF-β1 stimulation in osteoclasts also reportedly induced the expression of LIF via Smad and nuclear factor-kappa B (NF-κB) pathways^34^. We therefore examined whether MAPK and NF-κB signaling pathways are involved in the RANKL-induced expression of LIF in osteoclasts. After the formation of osteoclasts, cell cultures were treated with each pathway-specific inhibitor for 24 h in the presence of M-CSF and RANKL. Both JNK and p38 MAPK inhibitors, but not ERK or NF-κB inhibitors, significantly suppressed *Lif* expression in RANKL-treated osteoclast cultures (Fig. 6D). These data suggest that RANKL stimulation in osteoclasts induces LIF expression via JNK and p38 MAPK pathways.

## Discussion

Here, we demonstrated that osteoclasts are abundant in trabecular bone and reduce sclerostin expression. Administration of an anti-RANKL antibody to C57BL/6 mice reduced the number of osteoclasts and LIF-positive cells, markedly increased the sclerostin-positive osteocytes in trabecular bone, and suppressed Wnt/β-catenin signal transduction and bone formation. Furthermore, *Rankl*^+/−^ mice exhibited fewer LIF-positive osteoclasts and an increased number of sclerostin-positive osteocytes in trabecular bone. *Rankl*^+/−^ mice exhibited fewer β-catenin-positive cells and reduced bone formation in trabecular bone. These results suggested that LIF-positive osteoclasts facilitated bone formation through the reduction of sclerostin expression in trabecular bone. In addition, RANKL signal increased LIF expression in cultured osteoclasts. Thus, the reduction of sclerostin expression by osteoclasts in the vicinity of osteocytes may play a key role in the high turnover rate in trabecular bone.

Mechanical loading is considered to be the most important factor for suppressing the expression of sclerostin^35,36^. Analysis of a mouse tibial axial compression loading model revealed that the strain-induced bone adaptation of cortical bone was similar to that of the trabecular bone^37^, indicating that sclerostin expression in cortical bone is similar to that in trabecular bone. However, we and others^27^ found that sclerostin expression in trabecular bone was lower than that in cortical bone. These findings suggest that factors other than mechanical loading suppress sclerostin expression in trabecular bone. We examined whether there is a negative correlation between osteoclast number and the sclerostin expression in trabecular bone. The number of osteoclasts on trabecular bone in diaphysis (area 1 mm away from metaphysis) was lower than that in metaphysis. However, the number of sclerostin-positive cells in trabecular in the diaphysis was comparable to that in the metaphysis (data not shown). These results suggest that sclerostin expression does not simply exhibit the negative correlation with the number of osteoclasts. In fact, *Rankl*^+/−^ mice exhibited higher expression of sclerostin even though the number of osteoclasts did not markedly decrease. Thus, not only osteoclast number but also the activity of osteoclasts may be critical for suppressing the expression of sclerostin. Based on the observation of bone sections stained for TRAP activity (Fig. 1E) and our previous results^8^, we hypothesized that osteoclasts reduce sclerostin expression in trabecular bone under physiological conditions. Furthermore, we substantiated here that the reduction of sclerostin expression by osteoclasts is important for bone formation in trabecular bone.

Maturation of osteocytes is considered to regulate the expression of sclerostin. Mature osteocytes strongly express sclerostin in cortical bone^38^. Since trabecular bone is thinner and remodeled more actively compared to cortical bone, the osteocytes in trabecular bone may be immature compared to those in cortical bone. Mechanical loading is another important regulator of sclerostin expression. Mechanical unloading elevated sclerostin expression in trabecular bone^39^ as well as cortical bone^39,40^. Therefore, these factors reduce sclerostin expression in trabecular bone under physiological conditions. The suppression of bone resorption by anti-RANKL antibody elevated sclerostin expression in trabecular bone even though the morphological features of the trabecular bone still remained almost unchanged. Thus, the expression of sclerostin may be strictly regulated by factors including them in trabecular bone as well as cortical bone. However, the suppression of bone resorption by anti-RANKL antibody increased bone mass in primary trabecular bone, which could reduce the strain density in response to normal loading. The mechanical loading decreases the sclerostin expression. It is possible that both reductions in osteoclast-derived LIF and the strain density may increase the sclerostin expression. Further analyses are required to clarify the mechanism by which sclerostin expression was upregulated in osteocytes with their maturation.

The mechanical unloading by immobilization reportedly increased sclerostin expression in osteocytes^39^ and then bone resorption^40^. The enhanced bone resorption does not increase bone formation. These findings suggest that the uncoupling of bone formation with resorption is caused by the mechanical unloading since the mechanical unloading increases the sclerostin expression. It is likely that osteoclasts cannot suppress the sclerostin expression in this situation. In contrast, bone resorption enhances bone formation though the suppression of sclerostin expression during physiological bone remodeling.

Serum sclerostin levels were reportedly increased in humans with aging^41,42^. The expression of sclerostin in mice was reportedly observed at 4 weeks of age^43^ and increased until 9 months old of age and then decreased at 21 months of age^44,45^. The number of osteoclasts in bone was reportedly increasedduring the growth period, and then tended to decrease with age^46,47^. Thus, these previous reports support in part our concept that osteoclasts suppress sclerostin expression in trabecular bone.

Osteoclast-secreted factors such as Cthrc1, CT-1, OSM, and S1P are reportedly implicated in the coupling mechanism between bone resorption and formation^1^. These factors possibly suppress the expression of sclerostin. We have previously shown that recombinant CTHRC1 was unable to reduce sclerostin expression in UMR106 cells and neutralizing antibodies against CT-1 and OSM failed to rescue osteoclast conditioned medium-suppressed effect on the expression of sclerostin in UMR106 cells^8^. These findings suggested that effects of these factors on suppression of sclerostin expression are weaker than that of LIF even though those factors are secreted from osteoclasts. However, in addition to LIF, several osteoclast-derived coupling factors have been reported^1,2^. Therefore, the inhibition of bone resorption may reduce bone formation by reducing the factors other than LIF. Further studies are necessary to clarify the involvement of these factors in sclerostin expression.

Previously, we reported that multinucleated bone-resorbing osteoclasts in *Opg*^*−*/*−*^ mice highly expressed LIF^8^. In this study, LIF-positive signals were observed only in the bone-resorbing osteoclasts of WT mice under physiological conditions (Figs. 2G, 4H). These findings suggest that bone-resorbing osteoclasts strongly express LIF. It has been previously reported that TGF-β1 promotes the expression of LIF with differentiation and activation of osteoclasts in vitro^34,48^. These data indicate that TGF-β1 released from the bone matrix during bone resorption leads to the upregulation of LIF expression in osteoclasts. Furthermore, we found that RANKL stimulation increased the expression of LIF in osteoclasts in culture experiments and analysis of *Rankl*^+/*−*^ mice. The RANKL signaling was reportedly important not only for osteoclast differentiation but also for its function^49^. We found that IL-1α, which activates bone resorption, promoted the expression of LIF in osteoclasts (data not shown). Osteocytes are the primary source of RANKL in bone^50^. As trabecular bone exhibits a mesh-like structure, osteocytes exist near the bone surface in the trabecular bone as compared with in cortical bone. In addition, membrane-bound RANKL, but not soluble RANKL, was reportedly involved in bone resorption in the periodontal disease model^51^. Membrane-bound RANKL is considered to more easily access osteoclasts in trabecular bone. Thus, RANKL signaling may be more activated in trabecular bone compared to in cortical bone, suggesting that RANKL stimulates osteoclasts to express LIF.

Our study using inhibitors demonstrated that RANKL signaling induced the expression of LIF in osteoclasts via JNK and p38 MAPK pathways. Previous report has shown that the TGF-β1 signaling activates Smad and NF-κB, and induces the expression of LIF in osteoclasts^34^. Thus, the expression of LIF in osteoclasts is controlled by different cytokine signals, suggesting that osteoclast-derived LIF likely plays a key role in bone remodeling. Further detailed analyses are required to address the mechanism by which osteoclasts express LIF.

Recombinant LIF activates bone remodeling in mouse calvarial organ culture^22^ and elevates RANKL expression in osteoblast-lineage cells^52^. These reports suggest that osteoclast-induced LIF further induces RANKL expression in osteoblast-lineage cells, including osteocytes. We found that osteoclasts reduced the expression of sclerostin and then increased the number of Wnt/β-catenin-positive cells on the surface of trabecular bone. Furthermore, *Axin2* mRNA expression in the whole tibia was increased. These observations suggested that osteoblast-lineage cells were activated by increased Wnt/β-catenin signals. Activated Wnt/β-catenin signals in osteoblast-lineage cells may increase OPG expression. Thus, activated osteoclasts may terminate bone resorption by inducing OPG expression in the surrounding osteoblast-lineage cells and osteocytes.

Clinical studies reported that treatment of patients with osteoporosis with bisphosphonate or anti-RANKL antibody (denosumab) maintains bone mass through inhibition of bone resorption and helps lower the fracture rate^9,53,54^. However, administration of antiresorptive agents rarely causes atypical fractures or antiresorptive agent-related osteonecrosis of the jaw (ARONJ)^55,56^. One of the causes may be the rapid deterioration of bone formation by antiresorptive agents^57^. Therefore, a means for suppressing bone resorption by maintaining formation is beneficial for the treatment of osteoporosis.

In conclusion, we demonstrated that many osteoclasts in trabecular bone expressed LIF and play a key role in the acceleration of bone formation through the suppression of sclerostin expression in osteocytes. Our study provides evidence that osteoclasts are important for maintaining bone morphology and mass in trabecular bone under physiological conditions. Based on previous reports and our present results, administration of LIF or an anti-sclerostin antibody to patients with reduced bone formation due to antiresorptive agents may be effective for maintaining bone formation through the depression of sclerostin expression.

## Materials and methods

### Mice and reagents

C57BL/6 male mice were purchased from Japan SLC (Shizuoka, Japan). *Rankl*^+/*−*^ mice (C57BL/6 background)^29^ were generated by one (J.M.P.) of the authors’ laboratories. *Rankl*^+/*−*^ and *Rankl*^+*/*+^ (WT) littermate male mice at 12 weeks of age were used in this study. For the animal experiments, the number of mice in each group was determined according to our previous reports^8,58^. No statistical method was used to predetermine the sample size. Mice used for experiments were randomly selected from each strain. The mice were housed in up to 5 mice per cage (12 cm × 15 cm × 23 cm). All mice were housed in a specific-pathogen-free facility at Matsumoto Dental University at 24 °C ± 2 °C and 50–60% humidity with a 12-h light/dark cycle, and had free access to sterilized water and a normal diet (CLEA, CE-2). According to the guidelines of the Animal Management Committee of Matsumoto Dental University, all procedures for animal care were approved and carried out. Recombinant GST-RANKL was purchased from Oriental Yeast (Tokyo, Japan) and the neutralizing antibody against mouse RANKL (clone OYC1)^28^ was obtained from Oriental Yeast. Recombinant human M-CSF (Leukoprol) was purchased from Kyowa Hakko (Tokyo). Inhibitors of JNK (SP600125), p38 (SB203580), ERK (PD98059), and nuclear factor-kappa B (NF-κB) (BAY11-7082) pathways were purchased from Fujifilm Wako Pure Chemical (Osaka, Japan).

To evaluate he effects of elimination of bone resorption on sclerostin expression in osteocytes, the anti-mouse RANKL antibody (5 mg/kg) was injected once subcutaneously (s.c.) into C57BL/6 mice at 10 weeks of age^28^, and then these mice were sacrificed on day 14.

### Generation of *Sost-Green* reporter mice

A RPCI-24-276O23 BAC clone containing the mouse *Sost* (sclerostin) gene was purchased from BACPAC Resource Center at Children’s Hospital Oakland Research Institute (Oakland, CA). The targeting vector was prepared by recombination approaches. The 184-bp *Sost* CDS in exon 1 was replaced with ZsGreen CDS, which was fused to 24 nucleotides after the *Sost* start codon. The 5′ homology arm containing the modified *Sost* exon 1 and 3′ arm were subcloned into the PL451 targeting vector. The targeting vector was digested with *Not*I and electroporated into the embryonic stem cell line R1 (129/Sv origin). Targeted clones were screened by G418 and verified by Southern blot analysis. Cloned ES cells were injected into C57BL/6 blastocysts to generate chimeric mice. Chimeric mice were bred with C57BL/6 and the offspring were backcrossed for more than 5 generations. Knock-in lines were maintained as heterozygous and used as *Sost*^*Green/*+^.

### Southern blotting analysis

Proteinase K digested tail DNA was extracted with phenol/chloroform and then precipitated with isopropanol. DNA was digested with the XbaI restriction enzyme then separated by electrophoresis. After transferring the DNA fragment onto a nitrocellulose membrane, the target fragments were detected by the DIG hybridization system (Sigma-Aldrich, St. Louis, MO, USA).

### Bone histomorphometry

For fluorescence labeling of mineralization sites in the anti-mouse RANKL antibody-treated or vehicle-treated C57BL/6, WT, and *Rankl*^+/*−*^ mice, calcein (Sigma-Aldrich; 10 mg/kg, s.c.) was injected at 72-h intervals on days 9 and 12. On day 14, five or six mice of each group were sacrificed at 12 weeks of age. Their tibiae were fixed in 70% ethanol and stained with Villanueva Goldner or toluidine blue to identify cellular components. The tibiae were embedded in glycol-methacrylate, sectioned, and were subjected to histomorphometric analysis. Images were also visualized using fluorescence microscopy. Bone histomorphometric analysis was outsourced to an external company to be performed in a blind fashion. According to the guidelines of the histomorphometry nomenclature committee of the American Society for Bone and Mineral Research, nomenclature and units were used^59^.

### Fluorescence imaging

After perfusion fixation, bone tissues were further fixed with 4% paraformaldehyde (PFA) at 4 °C for 24 h. The bone tissues were decalcified with 10% EDTA, immersed in 30% sucrose solutions at 4 °C, and then embedded in 5% carboxymethyl cellulose (Section-lab, Hiroshima, Japan) as described previously^60^. Sections with a thickness of 10 μm were prepared using Kawamoto’s film method^61^. Nuclear staining was performed by Hoechst 33342 (blue, Supplementary Fig. S5) from Dojindo laboratories (Kumamoto, Japan). Cryosections were observed under a fluorescence microscope. The *Sost-Green*-positive osteocytes in bone tissues were measured using ImageJ software (NIH, Bethesda, MD, USA). The region of interest (ROI) of trabecular bone was from the distal end of the growth plate in the distal femur to the center of femur. The ROI of cortical bone began 1 mm from the distal end of the growth plate in the distal femur. The ratio of the number of *Sost-Green*-positive cells/the number of nuclei (%) was calculated.

### Immunohistochemistry

The dissected tibiae and femora were fixed, decalcified, and then embedded in paraffin as described previously^8^. Histological sections were incubated with anti-alkaline phosphatase (ALP), tissue-non-specific antibody (ab108337, Abcam, Cambridge, UK), an anti-mouse LIF antibody (AB-449-NA, R&D Systems, Minneapolis, MN), anti-β-catenin antibody (ab32572, Abcam), or anti-mouse sclerostin antibody (AF1589, R&D Systems) at 4 °C overnight. Next, the sections were incubated with the horseradish peroxidase (HRP)-conjugated secondary antibody. The HRP-conjugated antibody was visualized using a DAB kit (Nichirei bioscience, Tokyo). Cells were counterstained with hematoxylin. The histologies of negative controls were shown (Supplementary Fig. S6). The bone area in trabecular bone was measured using ImageJ software (NIH). The ROI of trabecular bone was from the distal end of the growth plate in the distal femur to the center of femur. The ROI of cortical bone began 1 mm from the distal end of the growth plate in the distal femur. On DAB staining, the number of sclerostin-positive cells was measured by histomorphometry, and the ratio of the number of sclerostin-positive osteocytes/bone area (N/mm^2^) was calculated. The numbers of LIF- and β-catenin-positive cells were also measured, and the ratio of the number of positive cells/bone surface (N/mm) was calculated. Histological sections were stained for tartrate-resistant acid phosphatase (TRAP, a marker of osteoclasts). For the TRAP-positive cells in Fig. 1E, the ROI of the box indicated by the dotted line was measured. The ROI of trabecular bone began from the distal end of the growth plate in the distal femur. The ROI of cortical bone began 0.2 mm from the distal end of the growth plate in the distal femur.

### Detection of bone metabolism markers in serum and bone marrow aspirates

Mouse serum and bone marrow aspirates were sampled and determined to ELISA analysis as described previously^62^. Serum levels of C-terminal crosslinked telopeptide of type-I collagen (CTX) and RANKL were measured by Ratlaps CTX- I EIA kit (immunodiagnostic systems, Boldon, United Kingdom) and ELISA (Quantikine Mouse TRANCE ELISA kit, R&D Systems), respectively. The serum activity of alkaline phosphatase (ALP) was measured using an ALP kit (Wako). Bone marrow aspirates were sampled from femora and tibiae in 0.5 ml of saline. Sclerostin amounts in bone marrow aspirates were measured by ELISA (Quantikine Mouse/Rat Sost ELISA kit, R&D Systems).

### BMM cultures

Mouse BMMs were prepared using osteoclast precursors as described previously^63^. BMMs were cultured in 24-well plates (7 × 10^4^ cells/well) in the presence of M-CSF (50 ng/ml) in αMEM (Sigma-Aldrich) containing 10% fetal bovine serum (FBS) (JRH Biosciences, Lenexa, KS). The BMMs were further cultured in the presence or absence of GST-RANKL (100 ng/ml), mouse GM-CSF (10 ng/ml; R&D Systems), mouse IL-4 (10 ng/ml; R&D Systems), or mouse IFN-γ (20 ng/ml; Peprotech, Rock Hill, NJ) with M-CSF (50 ng/ml) for 24 h. Total RNA was collected 24 h after stimulation.

### Osteoclast cultures

BMMs were cultured in 24-well plates (7 × 10^4^ cells/well) in the presence of GST-RANKL (100 ng/ml) and M-CSF (50 ng/ml) in αMEM (Sigma-Aldrich) containing 10% FBS. The BMM culture medium was changed day 2. In these cultures, osteoclasts were formed on day 3. Osteoclasts were further cultured in the presence or absence of GST-RANKL (1, 5, 25, or 100 ng/ml) with M-CSF (50 ng/ml) for 24 h. For inhibitor experiments, osteoclasts were cultured in the presence or absence of inhibitors of JNK (10 μM), p38 MAPK (10 μM), ERK (20 μM), or NF-κB (5 μM) pathways with GST-RANKL (100 ng/ml) and M-CSF (50 ng/ml) for 24 h. Total RNA was collected from these cultures on day 4.

### Real-time RT-PCR analysis

Cultured cells were lysed in the cultured dish using TRIzol Reagent (Thermo Fisher Scientific, Waltham, MA) to prepare total RNA. After flushing out bone marrow cells, the tibiae were homogenized in TRIzol using TissueLyser II (Qiagen, Hilden, Germany). Total RNA was isolated using RNA isolation kits (PureLink RNA mini kit, Thermo Fisher Scientific) and RNase-free DNase I (Qiagen). To synthesize first-strand cDNA, total RNA was reverse transcribed using oligo (dT)_12–18_ primers (Thermo Fisher Scientific) and ReverTra Ace (Toyobo, Osaka). The quantification of cDNA was performed by real-time RT-PCR using Fast SYBR Green (Thermo Fisher Scientific) and the StepOnePlus system (Thermo Fisher Scientific). The PCR reaction used following temperature profile: 95 °C for 20 s, followed by 40 cycles of 95 °C for 3 s and 60 °C for 30 s. The melting curve were confirmed in each PCR experiment. In Supplementary Table S1, the primer sequences used for real-time PCR analyses are listed. The levels of expression were calculated by a relative standard curve. As an internal control for normalization, *Gapdh* was used.

### Analysis of statistical significance

The results are expressed as the mean ± SD for three or more samples. In between two groups, the significance of differences was determined by the unpaired 2-tailed Welch’s *t* test. Among three or more groups, the significance of differences was determined by ANOVA with Scheffe’s test. Significant differences were considered as *P*-values less than 0.05.

## Supplementary information

Supplementary information.

## References

[1] Sims NA, Martin TJ (2015). Coupling signals between the osteoclast and osteoblast: How are messages transmitted between these temporary visitors to the bone surface?. Front. Endocrinol..

[2] Wein MN, Kronenberg HM (2018). Regulation of bone remodeling by parathyroid hormone. Cold Spring Harb. Perspect. Med..

[3] Suda T (1999). Modulation of osteoclast differentiation and function by the new members of the tumor necrosis factor receptor and ligand families. Endocr. Rev..

[4] O'Brien CA, Nakashima T, Takayanagi H (2013). Osteocyte control of osteoclastogenesis. Bone.

[5] Bucay N (1998). osteoprotegerin-deficient mice develop early onset osteoporosis and arterial calcification. Genes Dev..

[6] Mizuno A (1998). Severe osteoporosis in mice lacking osteoclastogenesis inhibitory factor/osteoprotegerin. Biochem. Biophys. Res. Commun..

[7] Nakamura M (2003). Osteoprotegerin regulates bone formation through a coupling mechanism with bone resorption. Endocrinology.

[8] Koide M (2017). Bone formation is coupled to resorption via suppression of sclerostin expression by osteoclasts. J. Bone Miner. Res..

[9] Reid IR (2010). Effects of denosumab on bone histomorphometry: The FREEDOM and STAND studies. J. Bone Miner. Res..

[10] Gatti D (2012). Sclerostin and DKK1 in postmenopausal osteoporosis treated with denosumab. J. Bone Miner. Res..

[11] Li X (2005). Sclerostin binds to LRP5/6 and antagonizes canonical Wnt signaling. J. Biol. Chem..

[12] Baron R, Kneissel M (2013). WNT signaling in bone homeostasis and disease: From human mutations to treatments. Nat. Med..

[13] Brunkow ME (2001). Bone dysplasia sclerosteosis results from loss of the SOST gene product, a novel cystine knot-containing protein. Am. J. Hum. Genet..

[14] Balemans W (2001). Increased bone density in sclerosteosis is due to the deficiency of a novel secreted protein (SOST). Hum. Mol. Genet..

[15] Li X (2008). Targeted deletion of the sclerostin gene in mice results in increased bone formation and bone strength. J. Bone Miner. Res..

[16] Li X (2009). Sclerostin antibody treatment increases bone formation, bone mass, and bone strength in a rat model of postmenopausal osteoporosis. J. Bone Miner. Res..

[17] Saag KG, Petersen J, Grauer A (2018). Romosozumab versus alendronate and fracture risk in women with osteoporosis. N. Engl. J. Med..

[18] Bellido T (2005). Chronic elevation of parathyroid hormone in mice reduces expression of sclerostin by osteocytes: A novel mechanism for hormonal control of osteoblastogenesis. Endocrinology.

[19] Galea GL (2011). Sost down-regulation by mechanical strain in human osteoblastic cells involves PGE2 signaling via EP4. FEBS Lett..

[20] Walker EC (2010). Oncostatin M promotes bone formation independently of resorption when signaling through leukemia inhibitory factor receptor in mice. J. Clin. Investig..

[21] Walker EC (2008). Cardiotrophin-1 is an osteoclast-derived stimulus of bone formation required for normal bone remodeling. J. Bone Miner. Res..

[22] Cornish J, Callon K, King A, Edgar S, Reid IR (1993). The effect of leukemia inhibitory factor on bone in vivo. Endocrinology.

[23] Poulton IJ, McGregor NE, Pompolo S, Walker EC, Sims NA (2012). Contrasting roles of leukemia inhibitory factor in murine bone development and remodeling involve region-specific changes in vascularization. J. Bone Miner. Res..

[24] Kramer I, Loots GG, Studer A, Keller H, Kneissel M (2010). Parathyroid hormone (PTH)-induced bone gain is blunted in SOST overexpressing and deficient mice. J. Bone Miner. Res..

[25] Leupin O (2007). Control of the SOST bone enhancer by PTH using MEF2 transcription factors. J. Bone Miner. Res..

[26] Li J (2017). Different bone remodeling levels of trabecular and cortical bone in response to changes in Wnt/beta-catenin signaling in mice. J. Orthop. Res..

[27] Hasegawa T (2013). Sclerostin is differently immunolocalized in metaphyseal trabecules and cortical bones of mouse tibiae. Biomed. Res..

[28] Furuya Y (2011). Increased bone mass in mice after single injection of anti-receptor activator of nuclear factor-kappaB ligand-neutralizing antibody: evidence for bone anabolic effect of parathyroid hormone in mice with few osteoclasts. J. Biol. Chem..

[29] Kong YY (1999). OPGL is a key regulator of osteoclastogenesis, lymphocyte development and lymph-node organogenesis. Nature.

[30] Hattersley G, Chambers TJ (1990). Effects of interleukin 3 and of granulocyte-macrophage and macrophage colony stimulating factors on osteoclast differentiation from mouse hemopoietic tissue. J. Cell. Physiol..

[31] Shioi A (1991). Interleukin 4 inhibits murine osteoclast formation in vitro. J. Cell Biochem..

[32] Takahashi N, Mundy GR, Roodman GD (1986). Recombinant human interferon-gamma inhibits formation of human osteoclast-like cells. J. Immunol..

[33] Alter J, Rozentzweig D, Bengal E (2008). Inhibition of myoblast differentiation by tumor necrosis factor alpha is mediated by c-Jun N-terminal kinase 1 and leukemia inhibitory factor. J. Biol. Chem..

[34] Ruan M, Pederson L, Bradley EW, Bamberger AM, Oursler MJ (2010). Transforming growth factor-{beta} coordinately induces suppressor of cytokine signaling 3 and leukemia inhibitory factor to suppress osteoclast apoptosis. Endocrinology.

[35] Robling AG (2008). Mechanical stimulation of bone in vivo reduces osteocyte expression of Sost/sclerostin. J. Biol. Chem..

[36] Lin C (2009). Sclerostin mediates bone response to mechanical unloading through antagonizing Wnt/beta-catenin signaling. J. Bone Miner. Res..

[37] Berman AG, Clauser CA, Wunderlin C, Hammond MA, Wallace JM (2015). Structural and mechanical improvements to bone are strain dependent with axial compression of the tibia in female C57BL/6 mice. PLoS ONE.

[38] Poole KE (2005). Sclerostin is a delayed secreted product of osteocytes that inhibits bone formation. FASEB J..

[39] Moustafa A (2012). Mechanical loading-related changes in osteocyte sclerostin expression in mice are more closely associated with the subsequent osteogenic response than the peak strains engendered. Osteoporos Int..

[40] Gaudio A (2010). Increased sclerostin serum levels associated with bone formation and resorption markers in patients with immobilization-induced bone loss. J. Clin. Endocrinol. Metab..

[41] Coulson J (2017). Circulating levels of dickkopf-1, osteoprotegerin and sclerostin are higher in old compared with young men and women and positively associated with whole-body bone mineral density in older adults. Osteoporos Int..

[42] Szulc P (2013). Correlates of bone microarchitectural parameters and serum sclerostin levels in men: The STRAMBO study. J. Bone Miner. Res..

[43] Watanabe T (2012). Increasing participation of sclerostin in postnatal bone development, revealed by three-dimensional immunofluorescence morphometry. Bone.

[44] Gardinier JD, Rostami N, Juliano L, Zhang C (2018). Bone adaptation in response to treadmill exercise in young and adult mice. Bone Rep..

[45] Piemontese M (2017). Old age causes de novo intracortical bone remodeling and porosity in mice. JCI Insight..

[46] Cao JJ (2005). Aging increases stromal/osteoblastic cell-induced osteoclastogenesis and alters the osteoclast precursor pool in the mouse. J. Bone Miner. Res..

[47] Glatt V, Canalis E, Stadmeyer L, Bouxsein ML (2007). Age-related changes in trabecular architecture differ in female and male C57BL/6J mice. J. Bone Miner. Res..

[48] Ota K (2013). Transforming growth factor beta 1 induces CXCL16 and leukemia inhibitory factor expression in osteoclasts to modulate migration of osteoblast progenitors. Bone.

[49] Jimi E (1999). Osteoclast differentiation factor acts as a multifunctional regulator in murine osteoclast differentiation and function. J. Immunol..

[50] Nakashima T (2011). Evidence for osteocyte regulation of bone homeostasis through RANKL expression. Nat. Med..

[51] Tsukasaki M (2018). Host defense against oral microbiota by bone-damaging T cells. Nat. Commun..

[52] Palmqvist P, Persson E, Conaway HH, Lerner UH (2002). IL-6, leukemia inhibitory factor, and oncostatin M stimulate bone resorption and regulate the expression of receptor activator of NF-kappa B ligand, osteoprotegerin, and receptor activator of NF-kappa B in mouse calvariae. J. Immunol..

[53] Brown JP (2009). Comparison of the effect of denosumab and alendronate on BMD and biochemical markers of bone turnover in postmenopausal women with low bone mass: A randomized, blinded, phase 3 trial. J. Bone Miner. Res..

[54] Cummings SR (2009). Denosumab for prevention of fractures in postmenopausal women with osteoporosis. N. Engl. J. Med..

[55] Marx RE (2003). Pamidronate (Aredia) and zoledronate (Zometa) induced avascular necrosis of the jaws: A growing epidemic. J. Oral. Maxillofac. Surg..

[56] Saad F (2012). Incidence, risk factors, and outcomes of osteonecrosis of the jaw: Integrated analysis from three blinded active-controlled phase III trials in cancer patients with bone metastases. Ann. Oncol..

[57] Jung J (2017). Short-term teriparatide and recombinant human bone morphogenetic protein-2 for regenerative approach to medication-related osteonecrosis of the jaw: A preliminary study. J. Bone Miner. Res..

[58] Okamoto M (2014). Noncanonical Wnt5a enhances Wnt/beta-catenin signaling during osteoblastogenesis. Sci. Rep..

[59] Dempster DW (2013). Standardized nomenclature, symbols, and units for bone histomorphometry: A 2012 update of the report of the ASBMR Histomorphometry Nomenclature Committee. J. Bone Miner. Res..

[60] Yang M (2017). Osteogenic factor Runx2 marks a subset of leptin receptor-positive cells that sit atop the bone marrow stromal cell hierarchy. Sci. Rep..

[61] Kawamoto T, Shimizu M (2000). A method for preparing 2- to 50-micron-thick fresh-frozen sections of large samples and undecalcified hard tissues. Histochem. Cell Biol..

[62] Tang Y (2009). TGF-beta1-induced migration of bone mesenchymal stem cells couples bone resorption with formation. Nat. Med..

[63] Sambandam Y (2016). Microgravity induction of TRAIL expression in preosteoclast cells enhances osteoclast differentiation. Sci. Rep..

