# Supplementary material for: Sclerostin expression in trabecular bone is downregulated by osteoclasts
Source: Sci Rep. 2020 Aug 13;10:13751. doi: 10.1038/s41598-020-70817-1 (PMC7426814; doi:10.1038/s41598-020-70817-1)
Supplement: Supplementary file 1 — Supplementary information. [file 41598_2020_70817_MOESM1_ESM.pdf]

Supplementary Information

Sclerostin expression in trabecular bone is downregulated by osteoclasts

Masanori Koide[1], Teruhito Yamashita[1], Kohei Murakami[2], Shunsuke Uehara[2], Keigo Nakamura[1], Midori Nakamura[2], Mai Matsushita[1], Toshiaki Ara[3], Hisataka Yasuda[4], Josef M. Penninger[5, 6], Naoyuki Takahashi[1], Nobuyuki Udagawa[1, 2], and Yasuhiro Kobayashi[1*]

[1]Institute for Oral Science, [2]Department of Biochemistry, [3]Department of Pharmacology, Matsumoto Dental University, 1780 Gobara, Hiro-oka, Shiojiri, Nagano 399-0781, Japan

[4]Nagahama Institute for Biochemical Science, Oriental Yeast Co., Ltd., 50 Kano-cho Nagahama, Shiga 526-0804, Japan

[5]Institute of Molecular Biotechnology of the Austrian Academy of Science (IMBA), Vienna Biocentre, Vienna, Austria

[6]Department of Medical Genetics, Life Science Institute, University of British Columbia, Vancouver, Canada

[*]**Corresponding author**:

Yasuhiro Kobayashi, D.D.S., Ph.D.

Division of Hard Tissue Research, Institute for Oral Science, Matsumoto Dental University, 1780 Gobara, Hiro-oka, Shiojiri, Nagano 399-0781, Japan

Phone +81-263-51-2238; Fax +81-263-51-2223

E-mail: yasuhiro.kobayashi@mdu.ac.jp

Supplementary Figure Legends

Supplementary Figure 1. Structure of the *Sost-Green* reporter gene

(A) Genomic structure at the *Sost* gene. (B) Southern blot analysis of wild-type (WT; +/+) and *Sost-Green* knock-in genotypes. The oligo probe for *Xba*I digests located downstream of the *Sost* locus detected a 6.1-kb fragment alone in wild-type and an additional 3.8 kb fragment for the *Sost-Green* knock-in locus in heterozygous mice.

Supplementary Figure 2. Embryonic expression of *Sost-Green*

Cryosections (t = 10 μm) were observed under a fluorescence microscope. Fluorescence (A, B, C, E, F, G) and bright field (A', B', C', D, E', F', G') images. During embryonic development, green-positive cells were hardly detected even in bony areas where calcified materials were observed (C; von Kossa staining). (A) E15.5, (B) E16.5, (C) E18.5. At three days after birth, strong green signals were observed in the bone cells of calvaria (E), limbs (F), and humerus midshaft cortical (G). Scale bars: 1 mm in (A)-(D), 0.5 mm in (E)-(G).

Supplementary Figure 3. Expression of sclerostin in the anti-RANKL antibody-treated C57BL/6 mice

Immunohistochemical analysis of sclerostin in the primary trabecular bone area of the femur from 12-week-old C57BL/6 male mice treated with vehicle (left panels) or the anti-RANKL antibody (right panels).

Supplementary Figure 4. Expression of DMP-1 and FGF-23 in *Rankl*$^{-/-}$ mice

(A) Histological analysis of alcian blue in the trabecular area of the femur from 12-week-old WT male mice (left panel) and *Rankl*$^{-/-}$ male mice (right panel). Cartilage matrix (blue). (B) Histological analysis of toluidine blue in the trabecular area of the femur from WT mice (left panel) and *Rankl*$^{-/-}$ mice (right panel). Cartilage matrix (purple). (C) Immunohistochemical

analysis of DMP-1 in the trabecular area of the femur from WT mice (left panel) and *Rankl*$^{-/-}$ mice (right panel). (D) Immunohistochemical analysis of FGF-23 in the trabecular area of the femur from WT mice (left panels) and *Rankl*$^{-/-}$ mice (right panels). Scale bar, 50 μm.

Supplementary Figure 5.

The higher magnifications image of nuclear staining. Scale bar, 50 μm.

Supplementary Figure 6. Negative controls in immunohistochemical analysis

(A) Immunohistochemical analysis of LIF in the trabecular area of the femur from WT mice. 1st antibody (left panels) and control goat IgG (right panels). (B) Immunohistochemical analysis of sclerostin in the trabecular area of the femur from WT mice. 1st antibody (left panels) and control goat IgG (right panels). (C) Immunohistochemical analysis of β-catenin in the trabecular area of the femur from WT mice. 1st antibody (left panels) and control rabbit IgG (right panels). (D) Immunohistochemical analysis of ALP in the trabecular area of the femur from WT mice. 1st antibody (left panels) and control rabbit IgG (right panels). Scale bar, 50 μm.

Supplementary Figure 7.

The full image of the Southern blot in Supplementary Figure 1B.

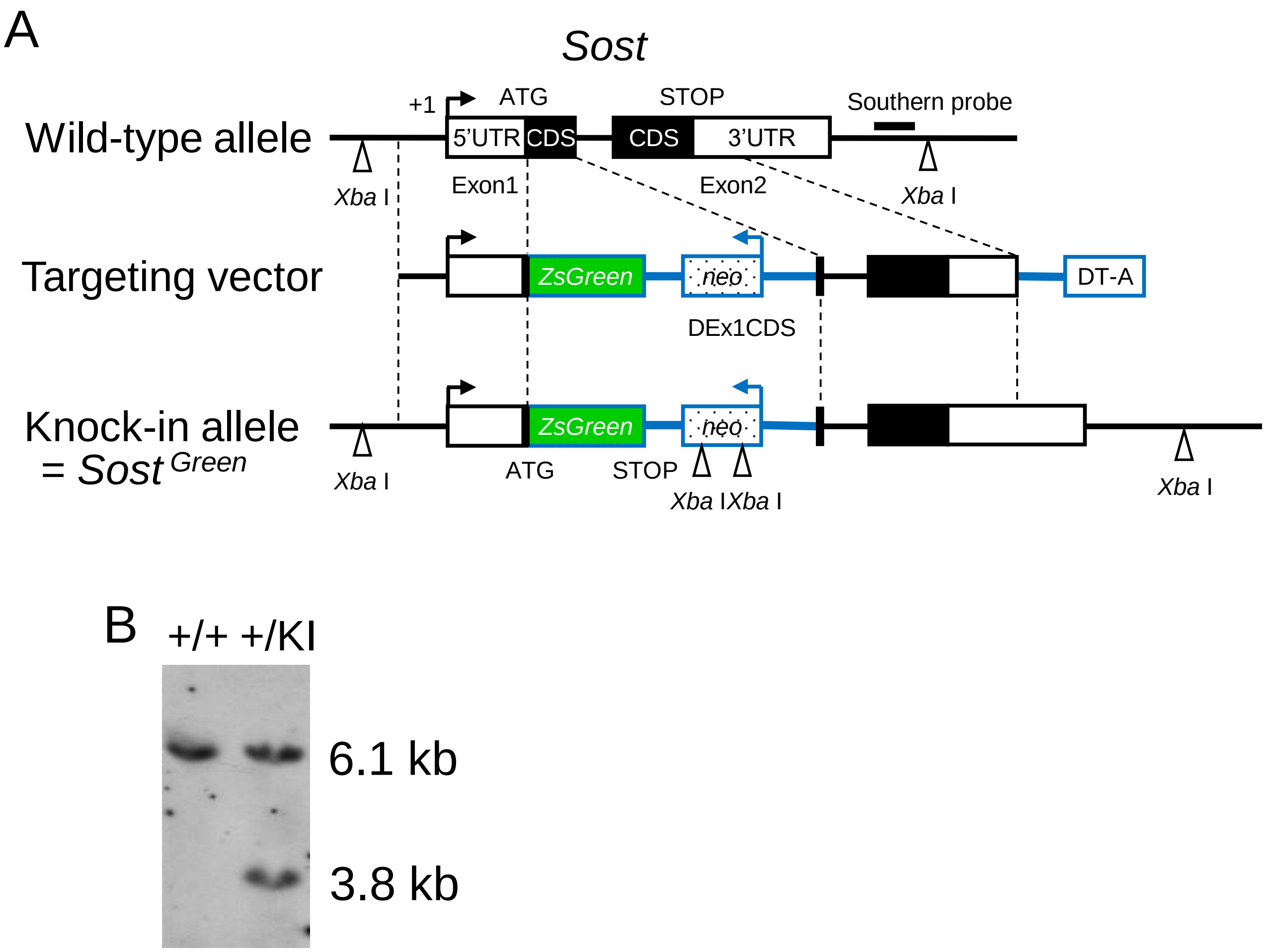

Supplementary Fig. S1.

Koide et al.

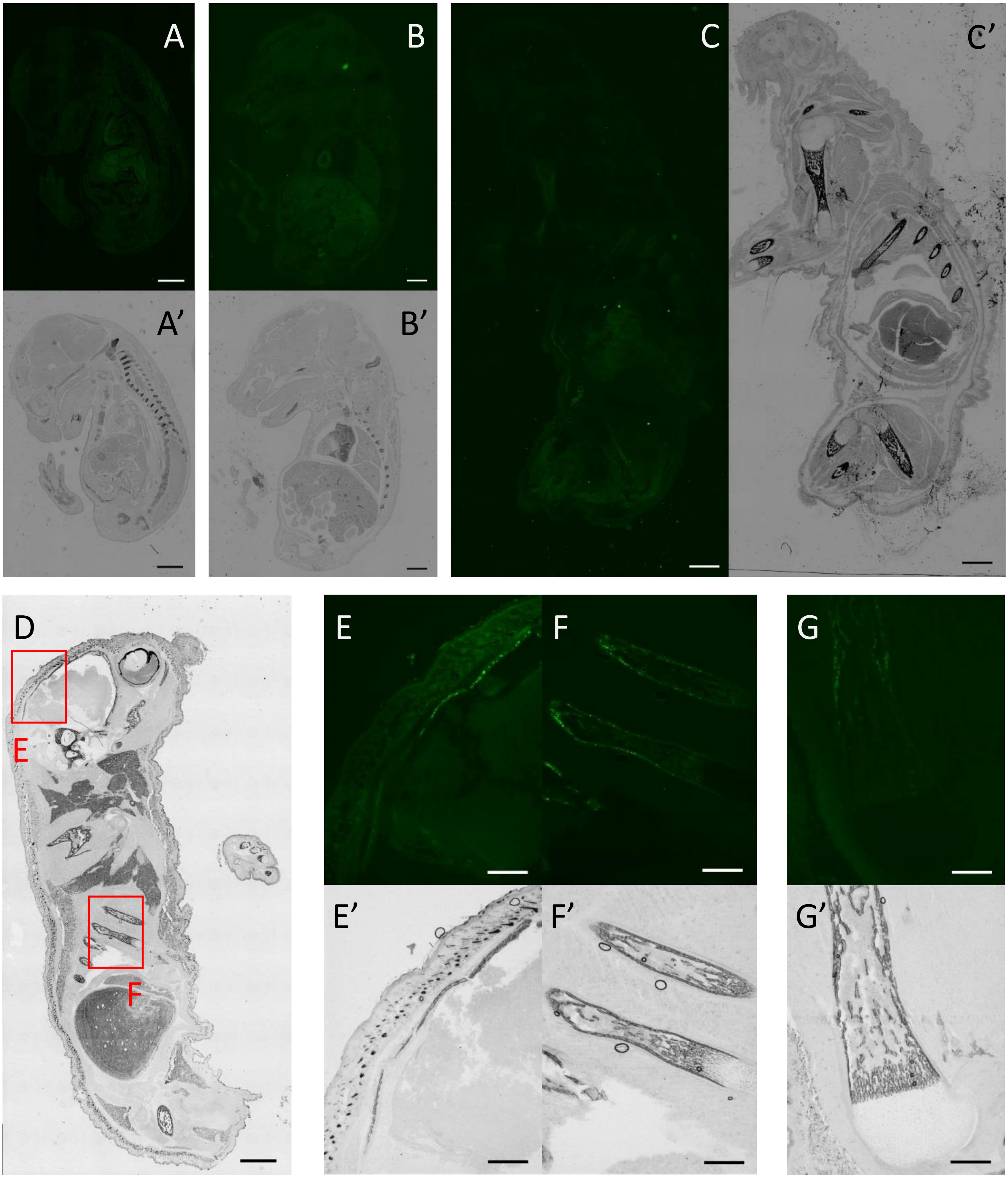

Supplementary Fig. S2.

Koide et al.

Sclerostin staining

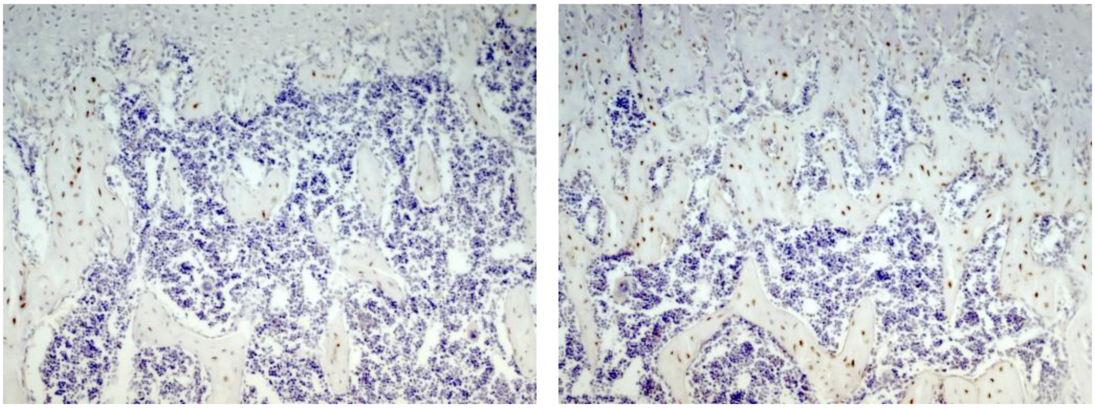

Vehicle

Anti-RANKL ab

Supplementary Fig. S3.

Koide et al.

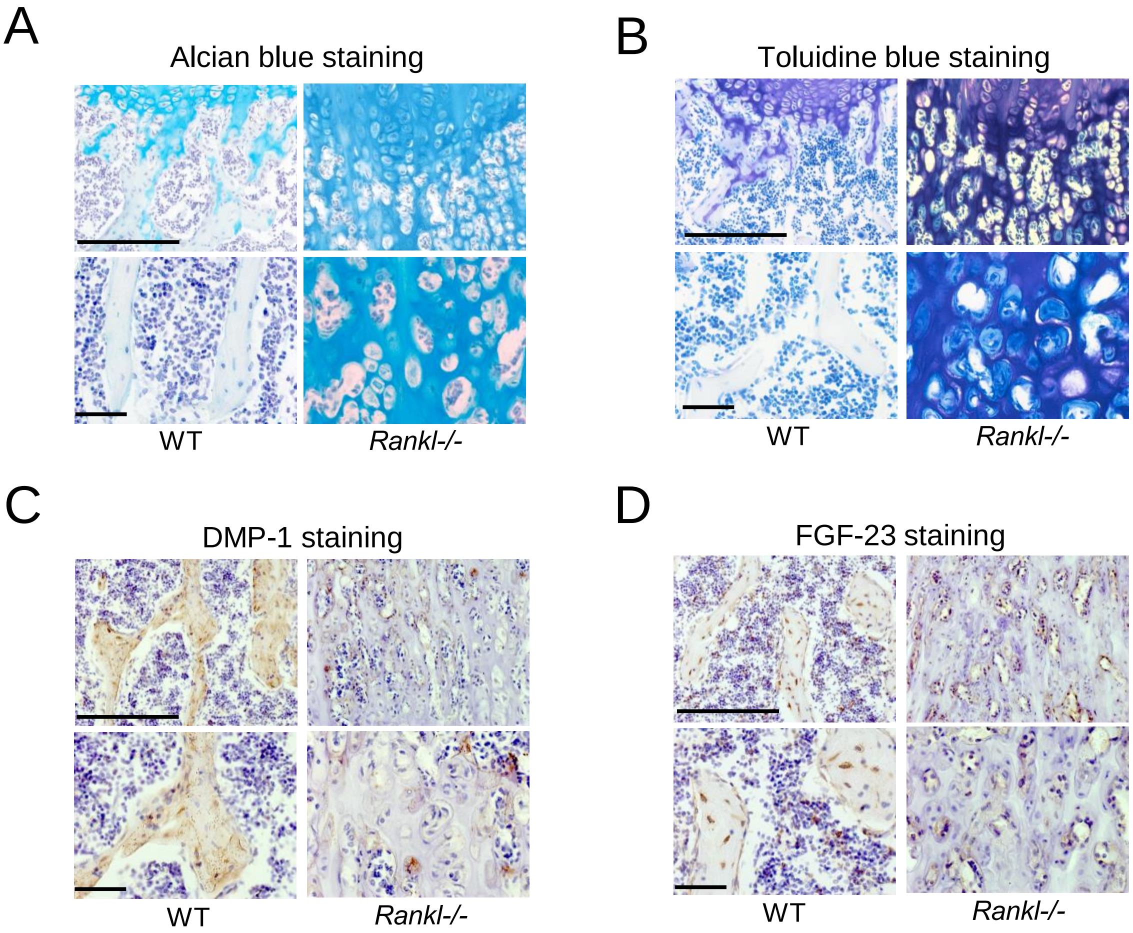

Supplementary Fig. S4.

Koide et al.

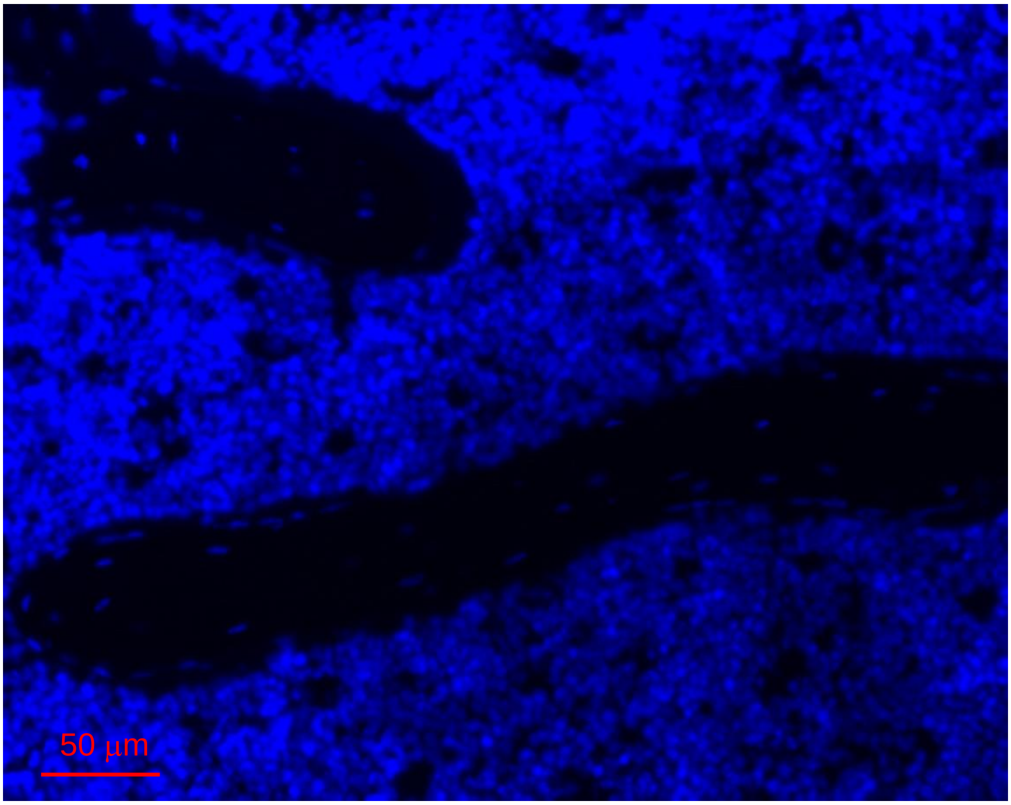

Supplementary Fig. S5. Koide et al.

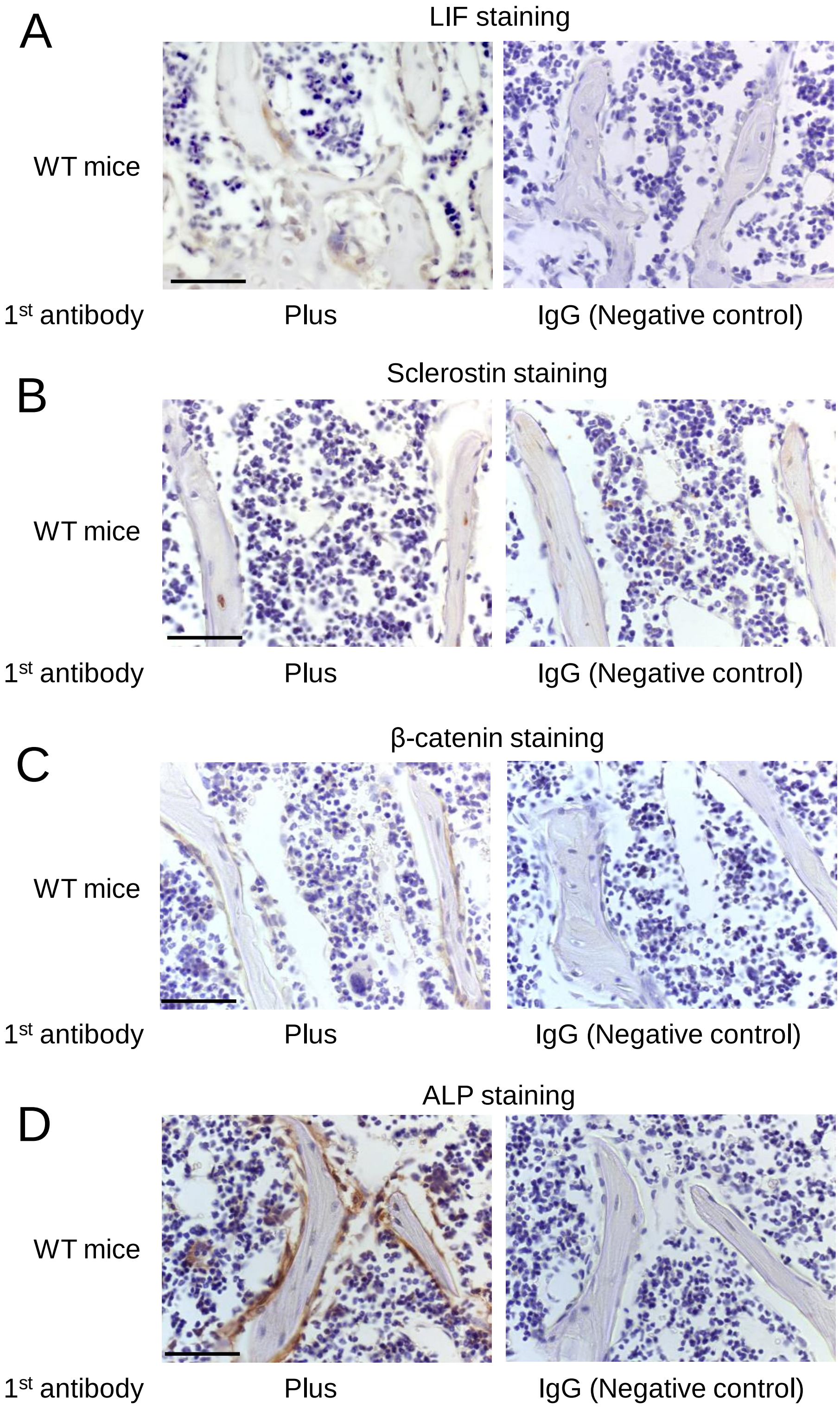

Supplementary Fig. S6. Koide et al.

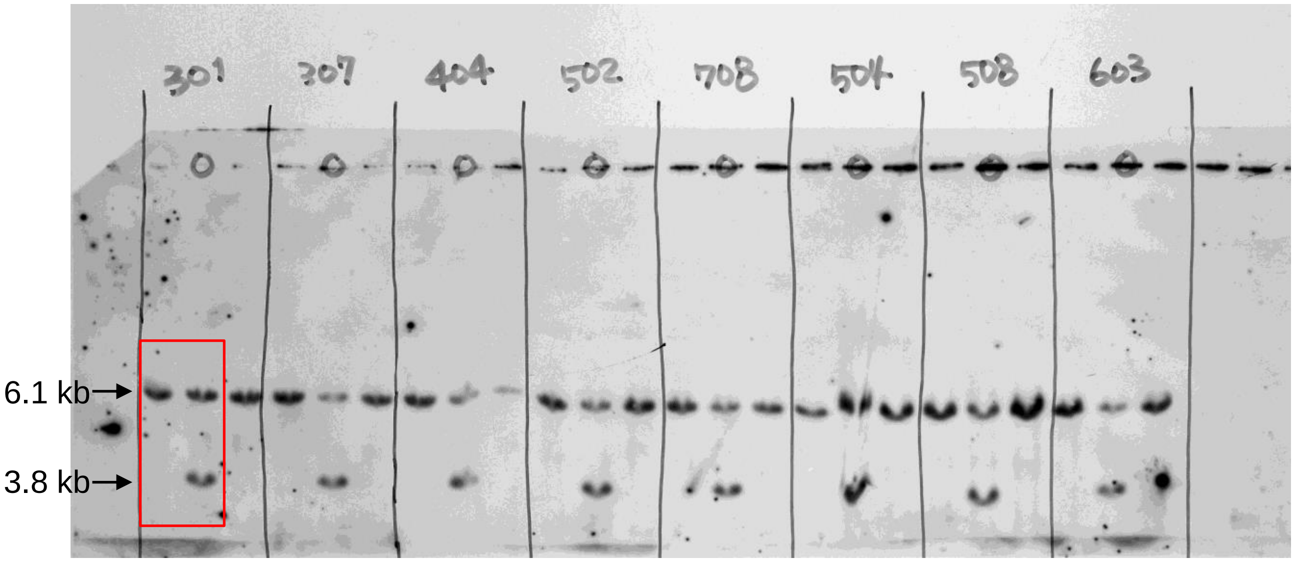

Supplementary Fig. S7. Koide et al.

Supplementary Table S1.

Primers for real-time PCR

	Forward	Reverse
Gapdh	TGTGTCCGTCGTGGATCTGA	TTGCTGTTGAAGTCGCAGGAG
Axin2	ATGTCCTGTCTGCCAGCGTTC	CAAGCACTAGCCAGTGGGTCAA
Lif	TTGCATGGTAGCGGCTTCAG	ATTTGTCACCCAAGGCCAAGTC
Acp5	TTGCGACCATTGTTAGCCACATA	TCAGATCCATAGTGAAACCGCAAG
Ctsk	CAGCAGAACGGAGGCATTGA	CTTTGCCGTGGCGTTATACATACA
Mmp9	GCCCTGGAACTCACACGACA	TTGGAAACTCACACGCCAGAAG
